# TNG961 Is a Selective Oral HBS1L Molecular Glue Degrader for the Treatment of *FOCAD-*Deleted Cancers

**DOI:** 10.1158/2159-8290.CD-26-0040

**Published:** 2026-04-19

**Authors:** Hilary E. Nicholson, Douglas A. Whittington, Frank J. Bruzzese, Katherine Lazarides, Lauren Catherine M. Martires, Matthew R. Tonini, Helena N. Jenkins, Minjie Zhang, Preksha Shahagadkar, Charlotte B. Pratt, Kimberly J. Briggs, Patrick McCarren, Alice W. Tsai, Madhavi Bandi, Chengyin Min, Alan Huang, Hongxiang Zhang, Samuel R. Meier, Binzhang Shen, Yi Yu, Colin Liang, Yong Liu, Teng Teng, John Zhang, Adam Crystal, William D. Mallender, Xinyuan Edward Wu, John P. Maxwell, Jannik N. Andersen

**Affiliations:** Tango Therapeutics, Boston, Massachusetts.

## Abstract

When tumor suppressor genes are lost through chromosomal deletion, the deletion of adjacent genes can generate therapeutic vulnerabilities. *MTAP* is frequently co-deleted with the chr9p21 tumor suppressor gene *CDKN2A*, creating a synthetic lethal dependency on protein arginine methyltransferase 5 (PRMT5). Telomeric to *MTAP* lies focadhesin (*FOCAD*), whose loss induces dependency on the HBS1-like translational GTPase (HBS1L)–protein pelota homolog (PELO) ribosome rescue complex for translational maintenance. *FOCAD* is deleted in ∼1 out of 3 *MTAP*-deleted cancers. We screened an immunomodulatory imide drug (IMiD)-focused diversity library and identified a weak hit that bound cereblon (CRBN), promoted HBS1L–CRBN–compound complex formation, and induced E3-ligase-dependent HBS1L ubiquitination and degradation. Guided by cryo-EM structures and proteome selectivity, we developed TNG961, a potent, selective HBS1L degrader that disrupts the HBS1L–PELO complex, inducing translational arrest, unfolded protein response activation, and growth inhibition in FOCAD-negative models. Oral administration of TNG961 regresses FOCAD-negative xenografts, including PRMT5 inhibitor-refractory models, establishing HBS1L degradation as a strategy to exploit FOCAD loss and supporting the clinical evaluation of TNG961 as a first-in-class precision oncology therapeutic.

**Significance:**

*FOCAD* deletion, frequently co-occurring with *MTAP*/*CDKN2A* loss, creates a synthetic lethal dependency on the HBS1L–PELO ribosome rescue complex. TNG961, a first-in-class molecular glue degrader of HBS1L, enforces translational arrest and drives tumor regressions in FOCAD-negative models, including PRMT5 inhibitor-refractory tumors, establishing a novel precision oncology strategy for chromosome 9p21 co-deletion contexts.

## Teaser

The HBS1L molecular glue degrader TNG961 exploits a translational dependency created by *FOCAD* loss, driving robust tumor regression and supporting a precision oncology strategy for chromosome 9p21 co-deleted cancers.

## Introduction

Deletion of tumor suppressor genes often results in collateral loss of neighboring genes, creating distinct context-specific cancer dependencies (1). Such “passenger” deletions may not be required for tumor initiation but are maintained during tumor progression, thereby creating opportunities for synthetic lethal therapeutic strategies. One prominent example is the co-deletion of *MTAP* with the tumor suppressor gene *CDKN2A* on chromosome 9p21. *MTAP* is co-deleted with *CDKN2A* in 10% to 15% of all human cancers, and inactivation of MTAP creates synthetic lethal dependencies on the methionine salvage pathway and on protein arginine methyltransferase 5 (PRMT5; refs. 2–8), leading to the clinical development of PRMT5 and MAT2A inhibitors for the treatment of *MTAP*-deleted tumors (9–17).

Telomeric to *MTAP* lies focadhesin (*FOCAD*), a gene that is co-deleted in approximately one third of *MTAP*-deleted cancers as part of the same 9p21 chromosomal loss event. *FOCAD* deletions occur across a broad range of tumor types, including cancers with high unmet medical need such as non–small cell lung cancer (NSCLC) and pancreatic cancer (Supplementary Fig. S1A). The *FOCAD* gene encodes the protein FOCAD, which plays a key role in maintaining mRNA quality control by stabilizing the superkiller (SKI) complex (18, 19). Together with the RNA exosome, the SKI complex mediates the degradation of aberrant mRNA species that would otherwise stall ribosomes and disrupt translational homeostasis (20–23). Consequently, loss of FOCAD destabilizes the SKI complex, resulting in the accumulation of defective transcripts and placing increased dependence on downstream ribosome rescue pathways to sustain protein synthesis (24, 25).

Multiple independent genetic studies show that FOCAD loss creates a selective dependency on the HBS1-like translational GTPase (HBS1L)–protein pelota homolog (PELO) complex, which functions in the rescue of stalled ribosomes. In the absence of SKI complex activity, this ribosome rescue pathway becomes essential for maintaining translational homeostasis, rendering FOCAD-negative cells exquisitely sensitive to the inactivation of HBS1L or PELO. Despite the strength of this compelling synthetic lethal interaction, small-molecule strategies to therapeutically exploit this vulnerability have not been described.

Targeted protein degradation has emerged as a powerful approach to drug otherwise intractable proteins by hijacking the ubiquitin–proteasome system. Molecular glue degraders, in particular, function by stabilizing novel protein–protein interactions between an E3 ubiquitin ligase and a neosubstrate, resulting in selective ubiquitination and degradation of the target protein. Cereblon (CRBN)-based molecular glues have shown success in the clinic, yet achieving neosubstrate selectivity, especially among closely related proteins, remains a central challenge (26). Given the high homology between HBS1L (also known as GSPT3) and the related translation termination factor and common immunomodulatory imide drug (IMiD)-based degrader neosubstrate GSPT1, targeting HBS1L through a molecular glue degrader modality is appealing, but identifying a selective degrader of HBS1L represents a stringent test of this modality.

Here, we report the discovery and preclinical characterization of TNG961 (27), a first-in-class molecular glue degrader that selectively targets HBS1L for CRBN-dependent degradation. Leveraging a dual cellular and biochemical screening strategy, followed by medicinal chemistry informed by cryo-EM analysis, we developed TNG961 as a potent, highly selective degrader that discriminates HBS1L from the closely related protein GSPT1. TNG961-mediated HBS1L degradation destabilizes the HBS1L–PELO ribosome rescue complex, triggering translational arrest, activation of the unfolded protein response (UPR), and loss of viability in FOCAD-negative cells while sparing FOCAD-positive counterparts. Across large-scale *in vitro* profiling, *FOCAD* loss emerged as the dominant biomarker of sensitivity, consistent with a synthetic lethal interaction driven by SKI complex dysfunction. *In vivo*, oral administration of TNG961 induced tumor regressions across multiple FOCAD-negative xenograft models from diverse histologies and retained activity in tumors progressing on PRMT5 inhibitor treatment. Together, these findings establish HBS1L degradation as a mechanistically distinct and biomarker-defined therapeutic strategy to exploit chromosome 9p21 co-deletions and support the clinical development of TNG961 as a precision oncology therapy with potential complementarity to existing MTAP-directed approaches.

## Results

### Genomic Context and Functional Impact of FOCAD Deletion in Cancer

**Figure 1.**
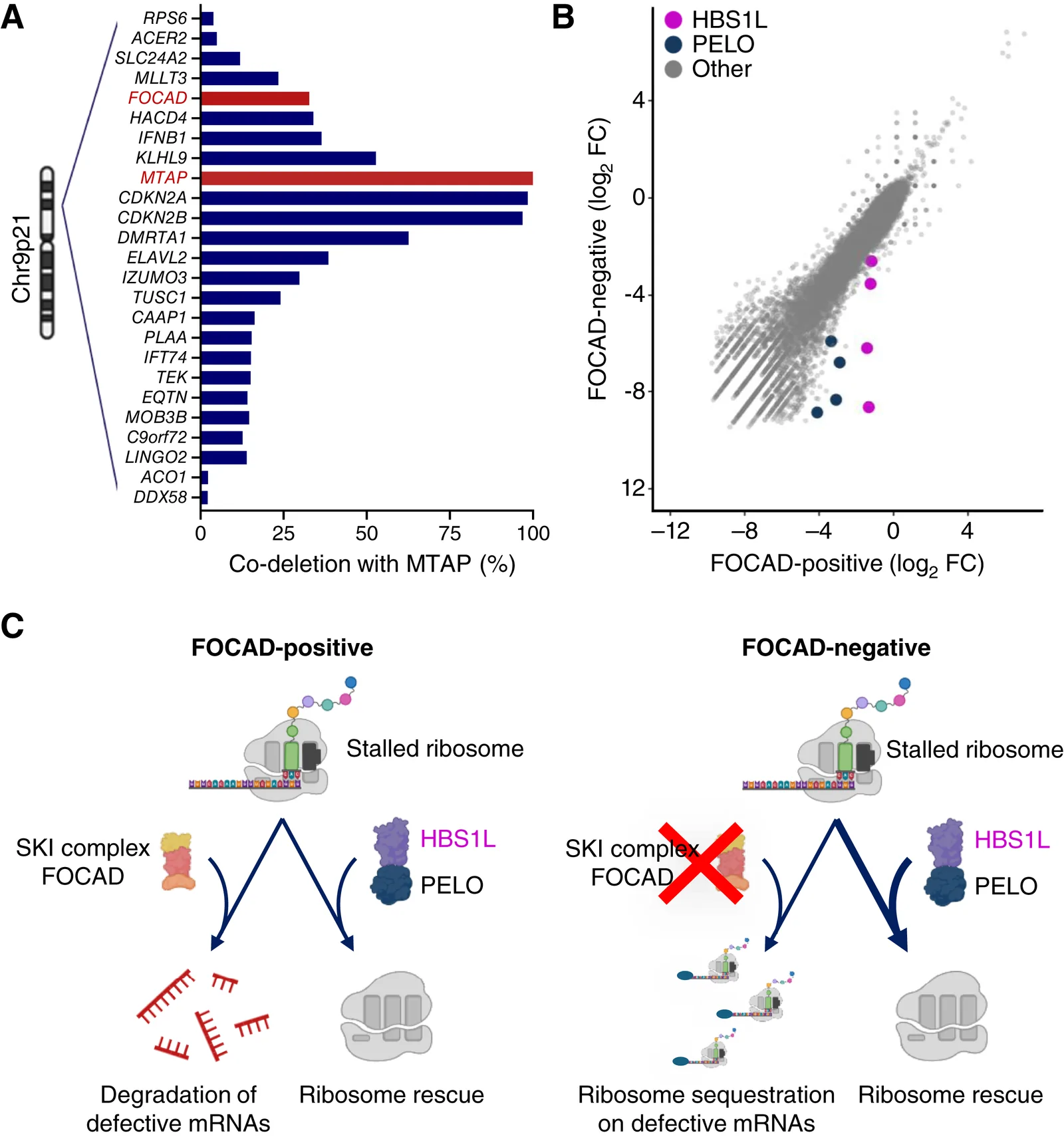
*FOCAD* deletion is frequent in cancer and confers hyperdependence on the HBS1L–PELO complex. **A,** Frequency of co-deletion with *MTAP* of chromosome 9p genes in proximity to *CDKN2A*. **B,** sgRNA-level effects from a genome-wide CRISPR screen in *FOCAD* isogenic MIAPACA2 cells. Cells expressing *FOCAD* cDNA (FOCAD-positive) or an empty vector control (FOCAD-negative) were infected with a genome-wide sgRNA library and cultured for 14 days prior to gDNA isolation and next-generation sequencing. Data are shown as log_2_ fold change (log_2_FC) in FOCAD-negative vs. FOCAD-positive cells. Raw CRISPR screening data previously reported (24). **C,** Model for the therapeutic hypothesis underlying the synthetic lethal interaction between *FOCAD* inactivation and the HBS1L–PELO complex. (Created in part with BioRender, Nicholson, H E. (2023) https://BioRender.com/ralcu3s).

Somatic deletion of tumor suppressor genes frequently extends beyond the driver locus. Pan-cancer analysis of TCGA datasets using cBioPortal reveals that approximately one third of *MTAP-*deleted cancers also harbor *FOCAD* deletions (Fig. 1A; refs. 5, 6, 28). Virtually all *FOCAD* deletions co-occur with loss of both *MTAP* and *CDKN2A*, with the latter being the tumor suppressor gene driving the co-deletion phenomenon. Overall, *FOCAD* alterations are present in ∼6% of all cancer cases, with deep deletions predominating (24). This includes ∼6% of NSCLC cases, highlighting a substantial patient population with high unmet medical need.

Based on functional genomic studies (DepMap analysis; refs. 19, 24, 25) and in-house CRISPR screens (24), we and others have shown that inactivation of FOCAD results in a marked dependency on the HBS1L-PELO ribosome rescue complex. HBS1L, a translational GTPase, and its binding partner PELO function together to resolve stalled ribosomes on nonstop or no-go mRNAs, thereby preventing translational collapse. This dependency is evident from genome-wide CRISPR screens using *FOCAD* isogenic cells (24), in which four independent sgRNAs targeting either HBS1L or PELO show potent dropout in FOCAD-negative cells relative to FOCAD-positive cells (Fig. 1B). Notably, knockout of HBS1L exhibits the strongest differential between FOCAD-positive and FOCAD-negative conditions, suggesting that selective pharmacologic targeting of HBS1L could provide a favorable therapeutic index compared with the more skewed pan-lethal effects observed upon PELO knockout (24).

Mechanistically, *FOCAD* encodes Focadhesion (FOCAD), a protein that stabilizes the SKI complex (18, 19), which, together with the exosome, degrades aberrant mRNAs that would otherwise stall ribosomes and disrupt translational maintenance (20–23). Accordingly, *FOCAD* loss disrupts SKI complex function, promoting the accumulation of defective transcripts and rendering cells hyperdependent on HBS1L-PELO-mediated ribosome rescue. Fig. 1C illustrates this therapeutic hypothesis: In FOCAD-positive cells, the FOCAD-stabilized SKI complex facilitates the degradation of defective mRNAs and limits reliance on ribosome rescue, whereas in FOCAD-negative cells, loss of SKI complex function leads to ribosome sequestration on aberrant mRNAs and a critical dependency on the HBS1L-PELO complex for translational maintenance. Consequently, inactivation of HBS1L-PELO is selectively lethal in the context of SKI complex disruption, providing a compelling therapeutic rationale for targeting HBS1L in *FOCAD*-deleted cancers.

### Parallel Cellular and Biochemical Screening Identifies Selective HBS1L Molecular Glues

**Figure 2.**
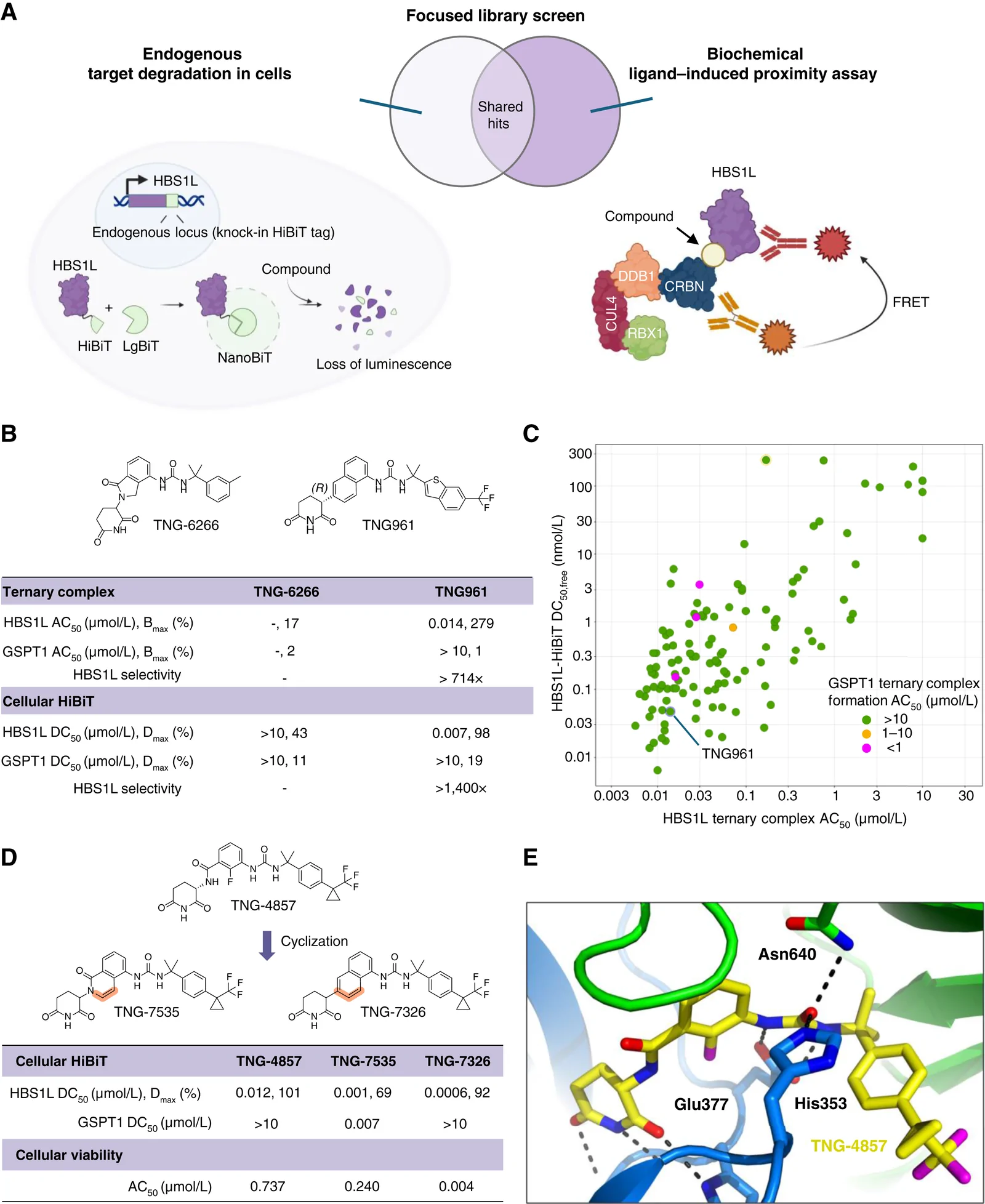
Hit finding strategy and discovery of molecular glues that drive CRBN-dependent ternary complex formation and selective degradation of HBS1L over GSPT1. **A,** Schematic of the parallel screening strategy used to identify HBS1L molecular glues. Left, HiBiT-based cellular assay using CRISPR knock-in of HiBiT at the endogenous HBS1L locus to enable high-throughput measurement of target degradation. Right, TR-FRET ternary complex assay to detect ligand-induced proximity between CRBN and HBS1L. Compounds active in both assays were prioritized. (Created in part with BioRender, Teng, T. (2026) https://BioRender.com/14wx5zd; Teng, T. (2026) https://BioRender.com/3lkesgf) **B,** Structures and activity summary for an initial screening hit TNG-6266 and optimized TNG961 in cellular degradation and ternary complex formation assays. AC_50_ is not reported when B_max_ < 50%; DC_50_ and AC_50_ values are corrected for media binding. **C,** Compound profiling across the HBS1L-HiBiT degradation assay and the HBS1L ternary complex formation (induced proximity) assay. Color indicates the degree of selectivity for HBS1L over GSPT1 with DC_50_ values corrected for media binding. **D,** Activity summary for TNG-4857, TNG-7535, and TNG-7326; DC_50_ and AC_50_ values are corrected for media binding. **E,** Binding of TNG-4857 (yellow) at the interface of HBS1L (green) and CRBN (blue) in the 2.6 Å resolution cryo-EM structure of the HBS1L–CRBN–DDB1 complex (PDB 11MR). Dashed lines indicate hydrogen bonds.

A dual screening strategy was implemented to identify compounds that both induce HiBiT-based cellular target degradation and promote ternary complex formation with CRBN in a biochemical assay (Fig. 2A). Because HBS1L was a pioneering molecular glue target and no commercial HBS1L-HiBiT knock-in cell line was available at the time, we generated cellular reagents by CRISPR engineering a HiBiT tag into the endogenous *HBS1L* locus in Promega LgBiT HEK cells, establishing a high-throughput reporter for the degradation of endogenous HBS1L rather than overexpressed constructs. To enforce selectivity upfront, we generated both N- and C-terminally tagged HBS1L knock-in lines and paired these with a commercially available 293T GSPT1-HiBiT knock-in line as an antitarget counterscreen. In parallel, we screened our proprietary molecular glue library in a time-resolved fluorescence resonance energy transfer (TR-FRET) ternary complex assay measuring compound-induced proximity between CRBN and either HBS1L or GSPT1.

The HiBiT cellular screens performed robustly (Z′ values >0.63) with high correlation between replicate plates (*r*^2^ = 0.662–0.891). Both CC-885 and CC-90009 served as positive controls for GSPT1-HiBiT degradation, whereas only CC-885 degraded HBS1L-HiBiT. Applying selection criteria of >20% target degradation with <20% viability impact yielded hit rates of 6.5% for HBS1L and 19% for GSPT1. In a parallel screen, the ternary complex formation assay was similarly robust (average *Z*′ = 0.61–0.72; DMSO coefficient of variance (CV) = 2.2–3.6%). Again, only CC-885 induced proximity between CRBN and both GSPT1 and HBS1L. Selecting for signals exceeding three CV above baseline produced hit rates of 0.58% (HBS1L) and 1.52% (GSPT1), with >70% reconfirmation in single-point follow-up assays.

From this screening campaign, TNG-6266 emerged as an initial hit with evidence of both cellular HBS1L degradation (*D*_max_ = 43% at 10 µmol/L) and ternary complex formation (*B*_max_ = 17%; Fig. 2B). Notably, TNG-6266 did not measurably degrade GSPT1 (DC_50_ > 10 µmol/L; *D*_max_ = 11%). These data provided an early indication that selective degradation of HBS1L was achievable and provided an encouraging starting point for the eventual development candidate TNG961 (Fig. 2B).

### Medicinal Chemistry

Medicinal chemistry efforts were guided by the primary screening assays (HiBiT degradation and ternary complex formation for both HBS1L and GSPT1; Fig. 2C), together with a 7-day viability assay in the NCIH1755 *FOCAD* isogenic cell line pair. Further ligand-based design was carried out to progress the series to the representative example TNG-4857 (Fig. 2D). Following the determination of a 2.6 Å resolution cryo-EM structure of TNG-4857 driving ternary complex formation between HBS1L and CRBN/DNA damage-binding protein (DDB1; Fig. 2E), several approaches were explored, including analogs wherein the ortho-fluorobenzamide was cyclized to form 6,6-bicyclic rings as in TNG-7535 and TNG-7326 (Fig. 2D). Interestingly, although TNG-7535 was more potent in the HBS1L degradation and viability assays compared with TNG-4857 (DC_50_ = 1 vs. 12 nmol/L; AC_50_ = 240 vs. 737 nmol/L), it also unexpectedly induced GSPT1 degradation (DC_50_ = 7 nmol/L). In contrast, the naphthyl-containing analog TNG-7326 further improved HBS1L ternary complex potency (AC_50_ = 4 nmol/L; 184-fold improvement vs. TNG-7535), while maintaining selectivity, with no detectable degradation of GSPT1 (DC_50_ > 10 µmol/L; Fig. 2D). Continued improvement of the naphthyl series led to TNG961, which was nominated as a clinical development candidate based on its *in vitro* and *in vivo* preclinical profile.

### TNG961 Rapidly Degrades HBS1L While Sparing GSPT1

**Figure 3.**
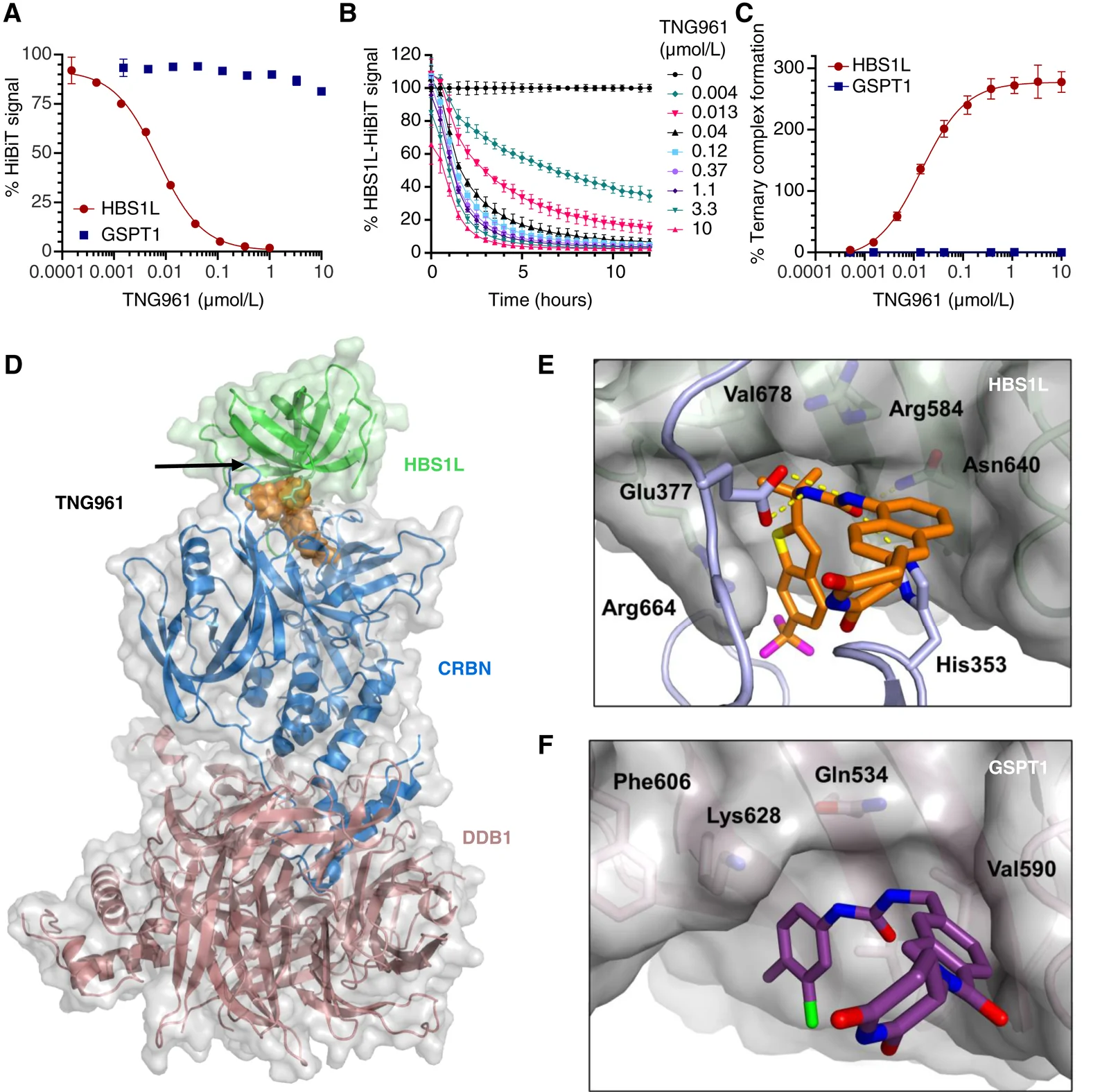
TNG961 mediates HBS1L–CRBN complex formation, leading to HBS1L degradation and FOCAD-dependent loss of viability. **A,** Cellular degradation of HBS1L and GSPT1 measured by HiBiT signal in 293T HBS1L-HiBiT and 293T GSPT1-HiBiT knock-in cell lines after 24 hours of treatment with the indicated concentrations of TNG961. Data are mean ± SD (*N* = 2 biological replicates; 2 technical replicates each). **B,** Live-cell degradation kinetics of HBS1L measured by HiBiT signal in 293T HBS1L-HiBiT knock-in cells over 12 hours following treatment with the indicated concentrations of TNG961. Data are mean ± SD (*N* = 2 biological replicates). **C,** TR-FRET ternary complex formation between CUL4A–RBX1–DDB1–CRBN and Avi-tagged HBS1L (red circles) or Avi-tagged GSPT1 (blue squares) in the presence of increasing concentrations of TNG961. Signal (%) is normalized to the CC-885–positive control well. Data are mean ± SD (*N* = 3 independent experiments; 2 technical replicates each). **D,** Cryo-EM structure (2.9 Å) of HBS1L (green) bound to the CRBN–DDB1 complex (blue and pink) in the presence of TNG961 (orange; PDB 10AY). **E,** Close-up surface view of TNG961 (orange) at the CRBN–HBS1L interface (PDB 10AY). The surface of HBS1L is depicted, and dashed lines indicate hydrogen bonds. **F,** Close-up surface view of CC-885 (purple) at the CRBN–GSPT1 interface (PDB 5HXB). The surface of GSPT1 is depicted.

In cellular screening assays, TNG961 treatment led to near-complete degradation of HBS1L (*D*_max_ = 98%; DC_50_ = 7 nmol/L at 24 hours), without affecting GSPT1-HiBiT levels, confirming selectivity for HBS1L over the closely related GSPT1 protein (Fig. 3A). To interrogate the degradation kinetics of TNG961, the HBS1L and GSPT1 HiBiT knock-in cell lines were used in live-cell assays. The HiBiT signal was measured every 30 minutes under nonlytic conditions following TNG961 treatment. Consistent with results from the lytic endpoint HiBiT assay, TNG961 induced dose-dependent degradation of HBS1L (Fig. 3B), whereas GSPT1-HiBiT showed no comparable modulation (Supplementary Fig. S1B). Maximal HBS1L degradation was reached within ∼4 hours at the highest concentration tested and remained suppressed throughout the 12-hour assay, supporting TNG961 as a rapid, potent, and selective degrader of HBS1L.

### TNG961 Selectively Drives CRBN–HBS1L Ternary Complex Formation and Ubiquitination

Using a TR-FRET ternary complex formation assay, TNG961 selectively promoted ternary complex formation between Cullin 4A (CUL4A)–RING-box protein 1 (RBX1)–DDB1–CRBN and HBS1L in a dose-dependent manner (AC_50_ value of 14.7 nmol/L), compared with an AC_50_ value >10 µmol/L for GSPT1 (Fig. 3C). By contrast, the nonspecific GSPT1 molecular glue degrader CC-885 (29–31) formed ternary complexes with both GSPT1 and HBS1L (Supplementary Fig. S1C). Notably, TNG961 achieved a higher maximal level of HBS1L ternary complex formation (*B*_max_) than CC-885, suggesting an enhanced propensity for complex formation and/or greater complex stability. These results underscore the ability of TNG961 to induce proximal association of HBS1L with a functional CRBN E3 ligase complex, with high selectivity over GSPT1.

To further assess selectivity, we developed an *in vitro* ubiquitination assay using purified proteins comprising the ubiquitination machinery (E1, E2, and E3 complex; refs. 32, 33) together with HBS1L or GSPT1. Under these conditions, both CC-885 and TNG961 promoted ATP-dependent polyubiquitination of HBS1L (Supplementary Fig. S1D). The presence of a molecular glue degrader molecule was essential for detectable polyubiquitination of either protein. In contrast, TNG961 did not induce polyubiquitination of GSPT1 (Supplementary Fig. S1E), further supporting selective CRBN–HBS1L complex formation.

### Structural Basis for HBS1L Recruitment and GSPT1 Selectivity

To define the molecular interactions driving the activity of TNG961, we solved the cryo-EM structure of full-length DDB1 bound to CRBN (amino acids 40–442) and HBS1L (amino acids 478–684) in the presence of TNG961 (Fig. 3D). The 2.9 Å resolution structure showed that the glutarimide-naphthalene moiety of TNG961 sits in the hydrophobic pocket of CRBN known to be occupied by the glutarimide moiety of other CRBN-targeted molecular glues (31, 34). The degron loop of HBS1L lines one side of this canonical pocket. The urea moiety of TNG961 forms hydrogen bonds with residues from both HBS1L and CRBN, and the trifluoromethyl-benzothiophene group sits in a hydrophobic pocket lined by residues from both HBS1L and CRBN (Fig. 3E). The guanidinium group of HBS1L residue Arg664 lies across the face of the benzothiophene ring in a cation-π stacking arrangement. Finally, the gem-dimethyl moiety of TNG961 sits adjacent to a β-sheet of HBS1L and forms van der Waals interactions with it.

Comparison with the reported crystal structure of CC-885 bound to the GSPT1–CRBN–DDB1 complex (Fig. 3F; ref. 31) indicates that CC-885 and TNG961 occupy overlapping regions within the CRBN binding pocket. Consistent with the known nonselective profile of CC-885, its binding mode is compatible with the recruitment of both GSPT1 and HBS1L. In contrast, differences in the surface topology of GSPT1 relative to HBS1L seem incompatible with the accommodation of TNG961, resulting in predicted steric clashes that are consistent with its experimentally observed selectivity profile. These structural observations provide a *post hoc* explanation for the selectivity of TNG961.

### TNG961 Induces CRBN-Dependent Endogenous HBS1L Degradation with Proteome-Wide Selectivity

**Figure 4.**
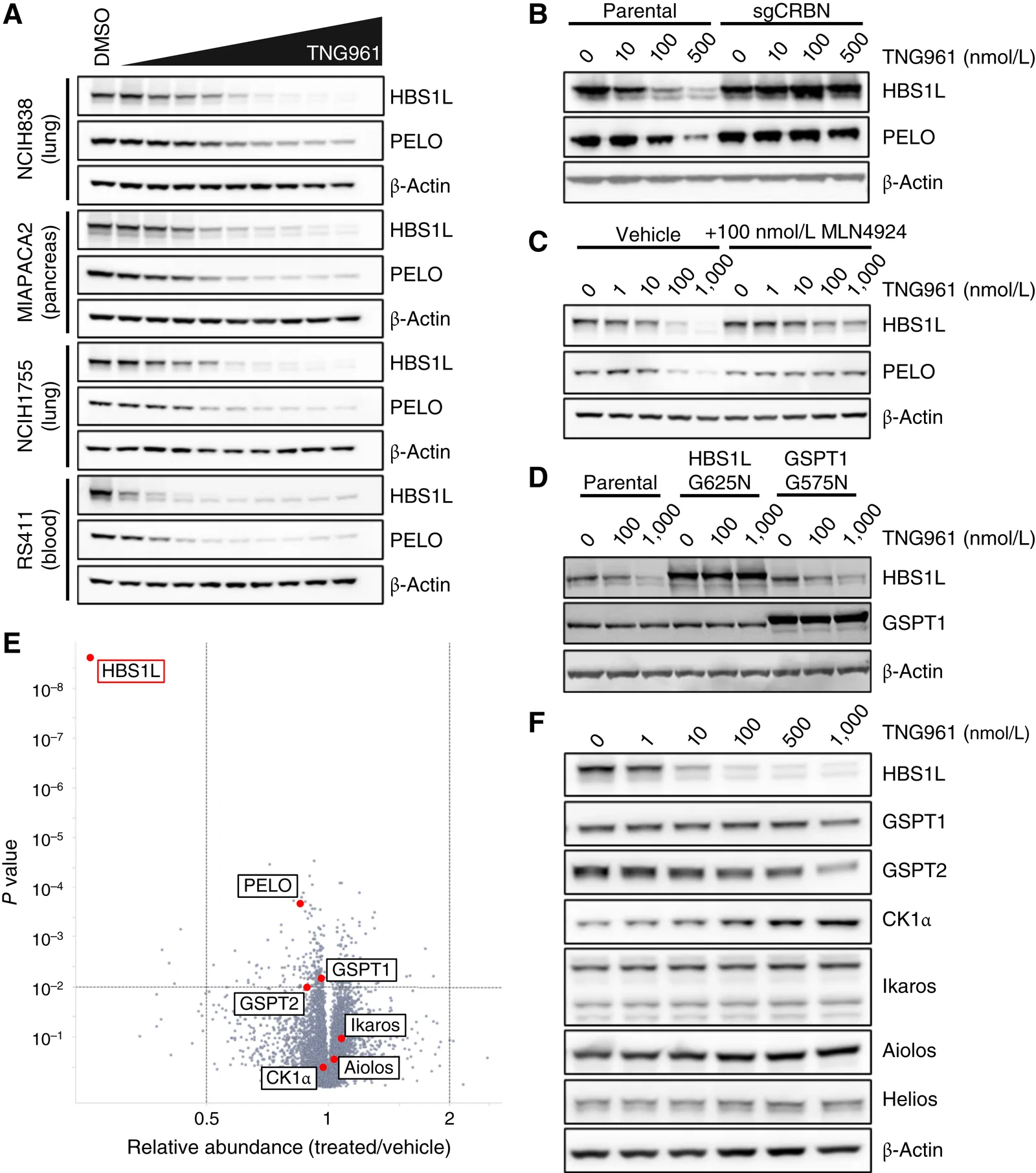
TNG961 induces potent, selective CRBN-dependent degradation of HBS1L. **A,** Endogenous HBS1L degradation and PELO destabilization measured by Western blot after 24 hours of treatment with TNG961 in the indicated cell lines. The top dose was 10 µmol/L, tested as a 9-point, 3-fold dilution series. Representative images are from *N* = 2 biological replicates. **B,** CRBN dependence of HBS1L degradation and PELO destabilization assessed by Western blot after 24 hours of treatment with the indicated concentrations of TNG961 in parental and CRBN knockout (sgCRBN) MIAPACA2 cells. Representative images are from *N* = 2 biological replicates. **C,** Neddylation dependence assessed by Western blot after 24 hours of treatment with TNG961 alone or in combination with 100 nmol/L MLN4924 in MIAPACA2 cells. Representative images are from *N* = 2 biological replicates. **D,** Degron-loop dependence assessed by Western blot after 24 hours of treatment with the indicated concentrations of TNG961 in parental MIAPACA2 cells and in engineered lines expressing either HBS1L G625N (covering endogenous HBS1L knockout) or GSPT1 G575N (covering endogenous GSPT1 knockout). Representative images are from *N* = 3 biological replicates. **E,** Global quantitative proteomics in MM.1s cells after 6 hours of treatment with 500 nmol/L TNG961 or DMSO. Data are shown as relative protein abundance (TNG961/DMSO); HBS1L, PELO, and common IMiD neosubstrates are annotated. **F,** Western blot analysis of common CRBN neosubstrates after 24 hours of treatment with TNG961 at the indicated concentrations. Representative images are from *N* = 2 biological replicates.

Consistent with the HiBiT assay results (Fig. 3A), TNG961 induced potent degradation of endogenous HBS1L across four cancer cell lines representing multiple lineages (NCIH838, MIAPACA2, NCIH1755, and RS411), as assessed by Western blot (Fig. 4A). Destabilization of the HBS1L binding partner PELO was also observed, consistent with prior genetic evidence demonstrating HBS1L-dependent PELO stability (24). CRBN knockout fully rescued TNG961-mediated degradation of HBS1L (Fig. 4B; Supplementary Fig. S1F), whereas cotreatment with the neddylation inhibitor MLN4924 (TAK924, pevonedistat; refs. 35, 36) partially restored HBS1L protein levels to ∼50% of DMSO, confirming the requirement for cullin-dependent E3 ligase ubiquitination (Fig. 4C).

CRBN interacts with certain neosubstrates through a conserved degron loop, exemplified by the G575N mutation in GSPT1 that abrogates CRBN-dependent degradation (31, 37) by the molecular glue degrader MRT-2359 (Supplementary Fig. S1G). Analogously, the introduction of the corresponding glycine mutation in the HBS1L degron loop (G625N) completely abrogated TNG961-induced HBS1L degradation (Fig. 4D), further confirming direct CRBN-mediated recognition of HBS1L.

To evaluate proteome-wide selectivity, global quantitative proteomics was performed in MM.1s cells, as this cell line expresses the majority of common IMiD-targeting neosubstrates. After 6 hours of treatment with TNG961, HBS1L was the most significantly altered protein (Fig. 4E; Supplementary DataSet S1). No additional proteins met predefined significance criteria (≥50% change in abundance; *P* < 0.01; >1 unique peptide; ≥10% sequence coverage) or reproduced at 24 hours. After 24 hours of treatment with TNG961, PELO was significantly depleted, consistent with HBS1L as the direct target and PELO destabilization occurring as a secondary consequence of HBS1L depletion (Supplementary Fig. S2A). If a less stringent *P* value criterion (*P* < 0.05) is applied, NUBPL was the only other protein identified with a significant change in relative abundance at 24 hours (Supplementary Fig. S2B, Supplementary DataSet S1) but with no known or predicted functional connections to HBS1L-PELO based on protein–protein interaction network analysis (STRING-db.org).

The transcription factor SALL4, a CRBN neosubstrate implicated in IMiD-associated teratogenicity (31, 38), is not expressed in MM.1s cells. Therefore, we assessed the impact of TNG961 treatment on SALL4 in the Kelly cell line (Supplementary Fig. S2C). Although SALL4 protein levels were decreased after treatment with thalidomide, pomalidomide, and lenalidomide, levels were unaffected by TNG961 at all doses tested. Other established CRBN neosubstrates (GSPT1, GSPT2, CK1α, Ikaros, Aiolos, and Helios) were similarly profiled and showed no significant degradation upon TNG961 treatment (Fig. 4F), supporting proteome-wide selectivity.

### TNG961 Selectively Impairs Growth of FOCAD-Negative Cells *In Vitro* and Leads to Tumor Regressions *In Vivo*

**Figure 5.**
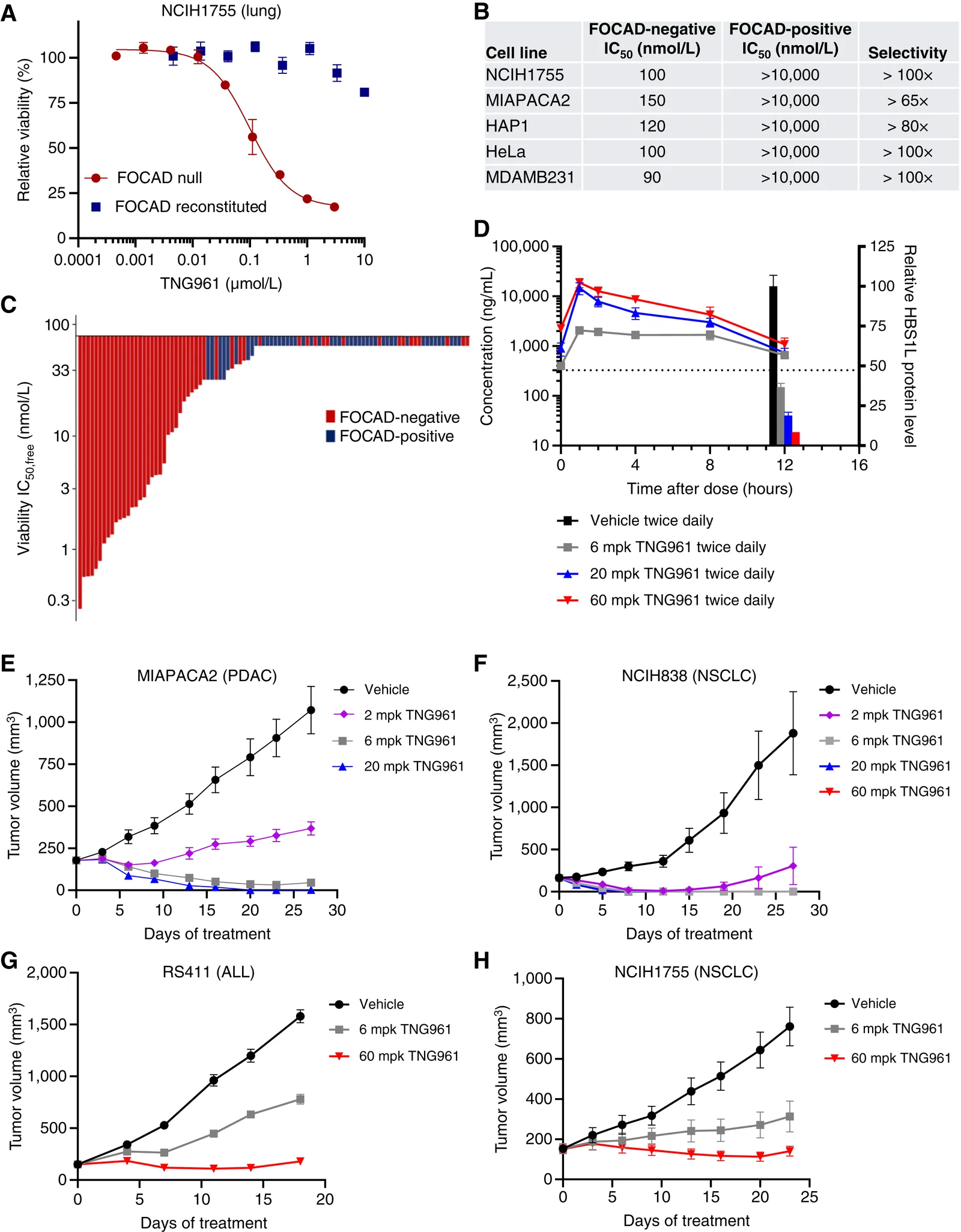
TNG961 selectively impairs the viability of FOCAD-negative cells *in vitro* and inhibits the growth of FOCAD-negative tumors *in vivo*. **A,** Relative viability of *FOCAD* isogenic NCIH1755 cells after 7 days of treatment with the indicated concentrations of TNG961, measured by CellTiter-Glo. Data are mean ± SD (*N* = 3 biological replicates). **B,** Relative viability of *FOCAD* isogenic cell line pairs from the indicated parental models after 7 days of treatment with TNG961, measured by CellTiter-Glo. HAP1 and HeLa are endogenously *FOCAD*-positive, and isogenic pairs were generated by *FOCAD* knockout; NCIH1755, MIAPACA2, and MDA-MB-231 are endogenously *FOCAD*-negative, and isogenic pairs were generated by re-expression of full-length *FOCAD* cDNA. Selectivity was calculated as potency in *FOCAD*-positive cells divided by potency in *FOCAD*-negative cells. Data are mean ± SD (*N* = 3 biological replicates). **C,** Potency of TNG961 across a panel of endogenously *FOCAD*-negative and *FOCAD*-positive cell lines after 7 days of treatment (maximum concentration 3 µmol/L), measured by CellTiter-Glo (viability assay) and adjusted to free potency to account for serum protein binding (5%–20% across culture conditions). Cell lines with free potency above the maximum concentration tested are plotted at the top dose. Data are mean ± SD (*N* = 2 technical replicates per model). **D,** Plasma exposure of TNG961 after 7 days of twice-daily dosing in MIAPACA2 xenograft–bearing mice and corresponding tumor pharmacodynamics assessed by Western blot 12 hours after the last dose (HBS1L protein levels shown as a fraction of the vehicle control band intensity). **E–H,** Tumor volume over time in mice bearing xenograft tumors (*N* = 8 mice/arm) from the indicated models treated orally with the indicated doses of TNG961 twice daily for the indicated duration. Data are mean ± SEM.

To evaluate the impact of TNG961 treatment on cell viability, we generated isogenic cell line pairs differing in *FOCAD* status. In the NSCLC cell line NCIH1755, which lacks endogenous *FOCAD*, re-expression of full-length cDNA restored FOCAD to protein levels comparable with those found in endogenous cell lines (Supplementary Fig. S3A). TNG961 inhibited the viability of FOCAD-negative NCIH1755 cells with a potency of 100 nmol/L, whereas FOCAD-reconstituted cells were unaffected at concentrations up to 10 µmol/L (Fig. 5A). This 100-fold selectivity window was recapitulated across additional *FOCAD* isogenic pairs spanning multiple lineages, including both FOCAD-negative cell lines re-expressing *FOCAD* cDNA (NCIH1775, MIAPACA2, MDAMB231) and FOCAD-positive cell lines (HeLa, HAP1) in which *FOCAD* had been knocked out (Fig. 5B; Supplementary Fig. S3B–S3E). The wide selectivity window between the growth inhibition of FOCAD-negative cells compared with FOCAD-positive cells suggests the potential for a large therapeutic index for TNG961.

### FOCAD Loss Is the Dominant Biomarker in Large-Scale Profiling

To further assess FOCAD status as a determinant of sensitivity, we profiled a panel of >90 parental cell lines comprising all reasonably obtainable FOCAD-negative models and a roughly matched set of FOCAD-positive, histology-matched controls, using a 7-day CellTiter-Glo assay (Fig. 5C). TNG961 showed strong potency only in FOCAD-negative cell lines, with most FOCAD-positive cell lines failing to respond at the highest dose tested. Cell lines with free potency values ≤ 10 nmol/L represented a broad spectrum of lineages, supporting FOCAD loss as the primary genetic driver of sensitivity rather than a lineage-specific dependency.

To further confirm both on-target activity and genomic context at scale, we conducted a 5-day multiplexed viability screen in collaboration with the PRISM Lab (Broad Institute of MIT and Harvard). Across >900 cell lines, the sensitivity profile of TNG961 correlated most strongly with *HBS1L* and *PELO* gene knockout signatures (Supplementary Fig. S4A). These data are consistent with our *in vitro* validation efforts and global proteomics studies, which identified HBS1L as the most significantly altered protein upon TNG961 treatment. Gene expression features most closely associated with sensitivity to TNG961 included *FOCAD* and *KLHL9*, another chromosome 9p21 gene located between *MTAP* and *FOCAD* (Fig. 1A) that is also frequently co-deleted with *FOCAD* (Supplementary Fig. S4B). Collectively, these findings support a strong FOCAD-based dependency within the broader chromosome 9p21 collateral-deletion context.

### Oral TNG961 Produces Exposure-Linked HBS1L Degradation and Tumor Regression

To translate these findings *in vivo*, we conducted pharmacokinetics and pharmacodynamics studies in MIAPACA2 xenografts. Oral dosing of TNG961 produced roughly dose-proportional plasma exposure (Fig. 5D), and after 7 days of twice-daily dosing, tumors harvested for Western blot analysis showed dose-dependent HBS1L degradation consistent with plasma exposures and the molecular glue degrader modality (Fig. 5D).

We next assessed whether tumor HBS1L degradation translated to antitumor activity. Mice implanted with FOCAD-negative cells were orally administered TNG961 twice daily and monitored for tumor growth. In MIAPACA2-derived xenografts, treatment led to dose-dependent tumor growth inhibition, with complete regressions observed in 3 out of 8 mice at 6 mg/kg and 7 out of 8 mice at 20 mg/kg (Fig. 5E). All doses of TNG961 were well tolerated, as inferred from body weight measurements (Supplementary Fig. S4C). Dose-dependent tumor growth inhibition was recapitulated in three additional FOCAD-negative models: NSCLC cell line NCIH838 (Fig. 5F), blood cancer cell line RS411 (Fig. 5G), and NSCLC cell line NCIH1755 (Fig. 5H). All models responded to TNG961 treatment, with significant tumor growth inhibition and good tolerability (Supplementary Fig. S4D–S4F). Together, these results demonstrate robust *in vivo* efficacy of TNG961 across multiple FOCAD-negative tumor types, supporting a histology-agnostic development strategy for *FOCAD*-deleted cancers.

### TNG961 Induces Translational Arrest and Activation of the Unfolded Protein Response in FOCAD-Negative, but Not FOCAD-Positive, Cells

**Figure 6.**
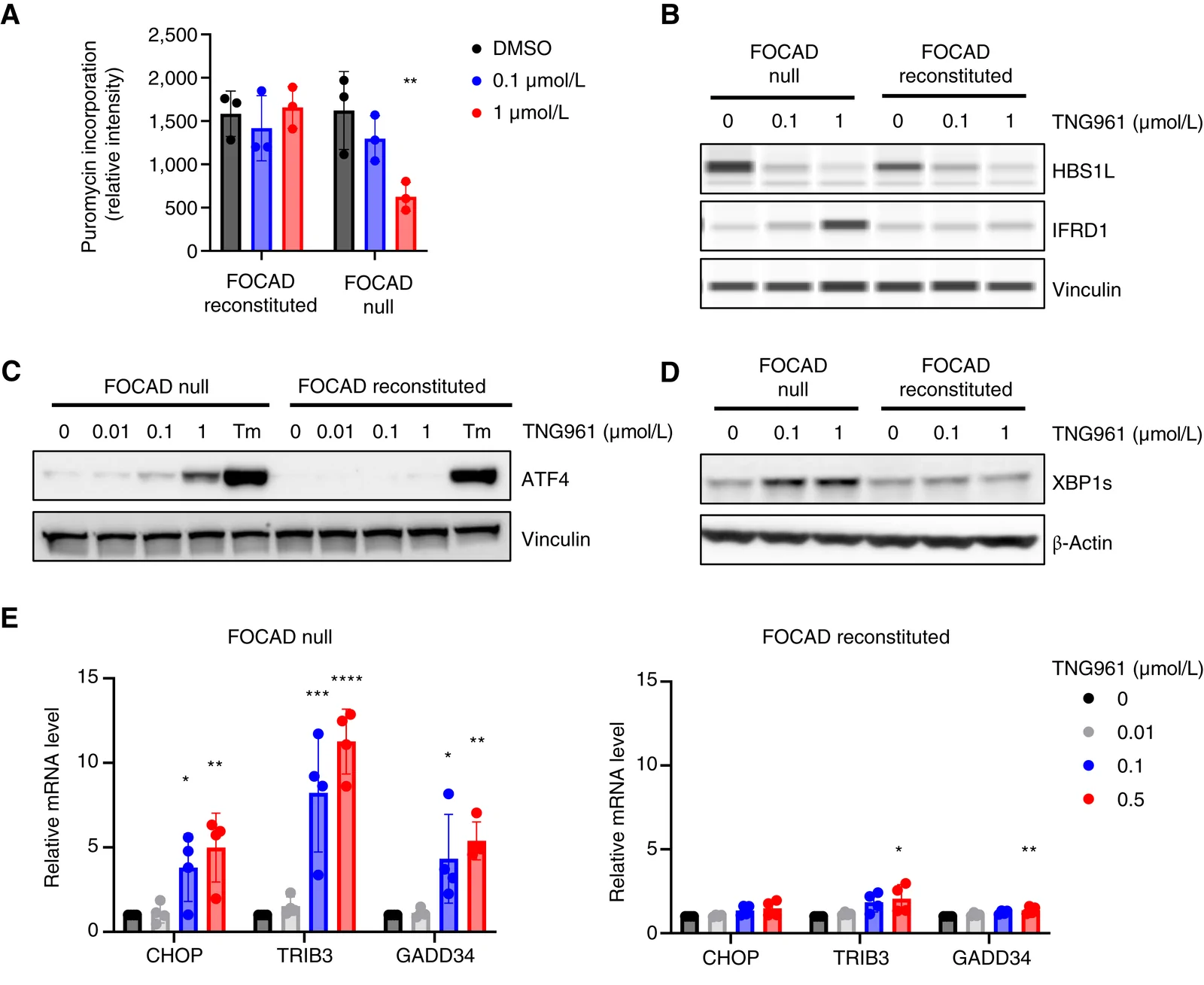
TNG961 induces translational arrest and activates ER stress and the UPR selectively in FOCAD-null cells. **A,** Translation assessed by puromycin incorporation in *FOCAD* isogenic MIAPACA2 cells after 48 hours of treatment with the indicated concentrations of TNG961. Puromycin immunoblot intensity was normalized to Ponceau staining to control for protein loading. Bars show mean ± SD (*N* = 3 biological replicates). **, *P* < 0.01 by two-way ANOVA with Sidak multiple comparisons test (vs. DMSO). Representative blots are shown in Supplementary Fig. S5A. **B,** HBS1L, IFRD1, and vinculin protein levels assessed by Simple Western in *FOCAD* isogenic MIAPACA2 cells after 48 hours of treatment with the indicated concentrations of TNG961. Representative images are from *N* = 2 biological replicates. **C,** ATF4 and vinculin protein levels assessed by Western blot in *FOCAD* isogenic MIAPACA2 cells after 48 hours of treatment with the indicated concentrations of TNG961. Tm, tunicamycin (positive control for ATF4 induction). Representative images are from *N* = 2 biological replicates. **D,** Spliced XBP1 (XBP1s) and actin protein levels assessed by Western blot in MIAPACA2 cells expressing a doxycycline (DOX)-inducible *FOCAD* cDNA (−DOX, FOCAD not expressed/FOCAD-null; +DOX, FOCAD expressed/FOCAD-reconstituted) after 72 hours of treatment with the indicated concentrations of TNG961. Representative images are from *N* = 2 biological replicates. **E,** qPCR analysis of CHOP, TRIB3, and GADD34 mRNA levels in *FOCAD* isogenic MIAPACA2 cells after 48 hours of treatment with the indicated concentrations of TNG961. Bars show mean ± SD (N = 4 biological replicates). *, *P* < 0.05; **, *P* < 0.01; ***, *P* < 0.001; ****, *P* < 0.0001 by one-way ANOVA with Dunnett multiple comparisons test (vs. DMSO). ANOVA *P* values (FOCAD null): CHOP *P* = 0.0044, TRIB3 *P* < 0.0001, GADD34 *P* = 0.0016; ANOVA *P* values (FOCAD reconstituted): CHOP *P* = 0.4290, TRIB3 *P* = 0.0320, GADD34 *P* = 0.0108.

Our data indicate that TNG961-mediated degradation of HBS1L selectively inhibits the growth of FOCAD-negative cells *in vitro* and *in vivo*. To elucidate the mechanism, we asked whether TNG961-mediated HBS1L degradation induces translational arrest in a FOCAD-dependent manner consistent with our therapeutic hypothesis (Fig. 1C). Using a puromycin incorporation assay to infer active translation, we observed that TNG961 significantly reduced puromycin incorporation into actively translated proteins in FOCAD-negative MIAPACA2 cells but not in FOCAD-reconstituted cells (Fig. 6A, Supplementary Fig. S5A). This change in translation is unlikely to be a consequence of decreased viability as reduced puromycin incorporation precedes viability defects by ∼5 days. The data support a model in which the activity of the HBS1L–PELO complex is required for translational homeostasis in the absence of FOCAD.

Ribosomes can become stalled with partially translated proteins on nonstop or no-go mRNA transcripts and depend on the HBS1L–PELO complex for rescue (39–41). In the absence of HBS1L-PELO activity, the accumulation of partially translated proteins can trigger endoplasmic reticulum (ER) stress and activation of the UPR (19, 24, 25, 42, 43). We hypothesized that this effect would disproportionately be amplified in cells that lack FOCAD due to SKI complex destabilization and persistent expression of aberrant mRNAs. Consistent with this model, TNG961 treatment selectively resulted in the upregulation of the ER stress marker IFRD1 in FOCAD-negative MIAPACA2 cells, with no induction in the isogenic FOCAD-reconstituted cells (Fig. 6B). Similar results (upregulation of IFRD1 in response to TNG961 treatment) were observed in *FOCAD* knockout, but not in wild-type, HeLa cells in which *FOCAD* is endogenously intact (Supplementary Fig. S5B). Likewise, we assessed stress-pathway engagement in MIAPACA2 xenograft tumors using samples from a 6-day PK/PD study. IFRD1 mRNA was significantly induced in TNG961-treated tumors relative to vehicle by qPCR (Supplementary Fig. S5C), consistent with our *in vitro* stress-response findings.

In addition to IFRD1 activation, we sought orthogonal lines of evidence to support the induction of ER stress and the UPR. Translation of ATF4 can be activated in parallel to IFRD1 and is a canonical feature of the ER stress response (18, 43). Consistent with UPR activation, TNG961 induced ATF4 protein expression in FOCAD-negative MIAPACA2 and HeLa cells, but not in their FOCAD-positive counterparts (Fig. 6C; Supplementary Fig. S5D). Another hallmark of ER stress is the splicing of the XBP1 transcription factor (44). During the UPR, the unspliced isoform of XBP1 is processed to generate an alternative active isoform (XBP1s). Consistent with our prior findings, TNG961 increased XBP1s levels in FOCAD-negative MIAPACA2 cells but did not induce XBP1 splicing in FOCAD-reconstituted cells (Fig. 6D).

Because ER stress signaling can drive a downstream transcriptional program linked to loss of viability, we next assessed UPR-associated gene induction in MIAPACA2 and HeLa *FOCAD* isogenic pairs. Each cell line pair was treated with TNG961, and qPCR was used to assess mRNA levels of CHOP, TRIB3, and GADD34 (Fig. 6E; MIAPACA2; Supplementary Fig. S5E; HeLa). Consistent with the protein level changes observed for IFRD1 and ATF4, we observed a ∼5- to 10-fold induction of CHOP, TRIB3, and GADD34 in FOCAD-negative cell lines but did not observe a similar impact in FOCAD-positive cell lines. These findings mirror published results in which *HBS1L* gene knockout elicits a similar transcriptional response in *FOCAD* isogenic cells (24). Together, these data support a model in which TNG961-induced degradation of HBS1L triggers translational arrest, ER stress, and UPR activation selectively in FOCAD-negative cells, contributing to impaired viability.

### TNG961 Induces Regression of Xenograft Tumors Progressing on PRMT5 Inhibitor Treatment

The studies above demonstrate robust monotherapy activity of TNG961 in FOCAD-negative tumors. Given the clinical promise of methylthioadenosine (MTA)-cooperative PRMT5 inhibitors and the fact that *FOCAD* deletion due to collateral *CDKN2A* loss is accompanied by *MTAP* co-deletion, we asked whether TNG961 could provide complementary activity in *MTAP/FOCAD* co-deleted tumors progressing on PRMT5 inhibitor therapy.

**Figure 7.**
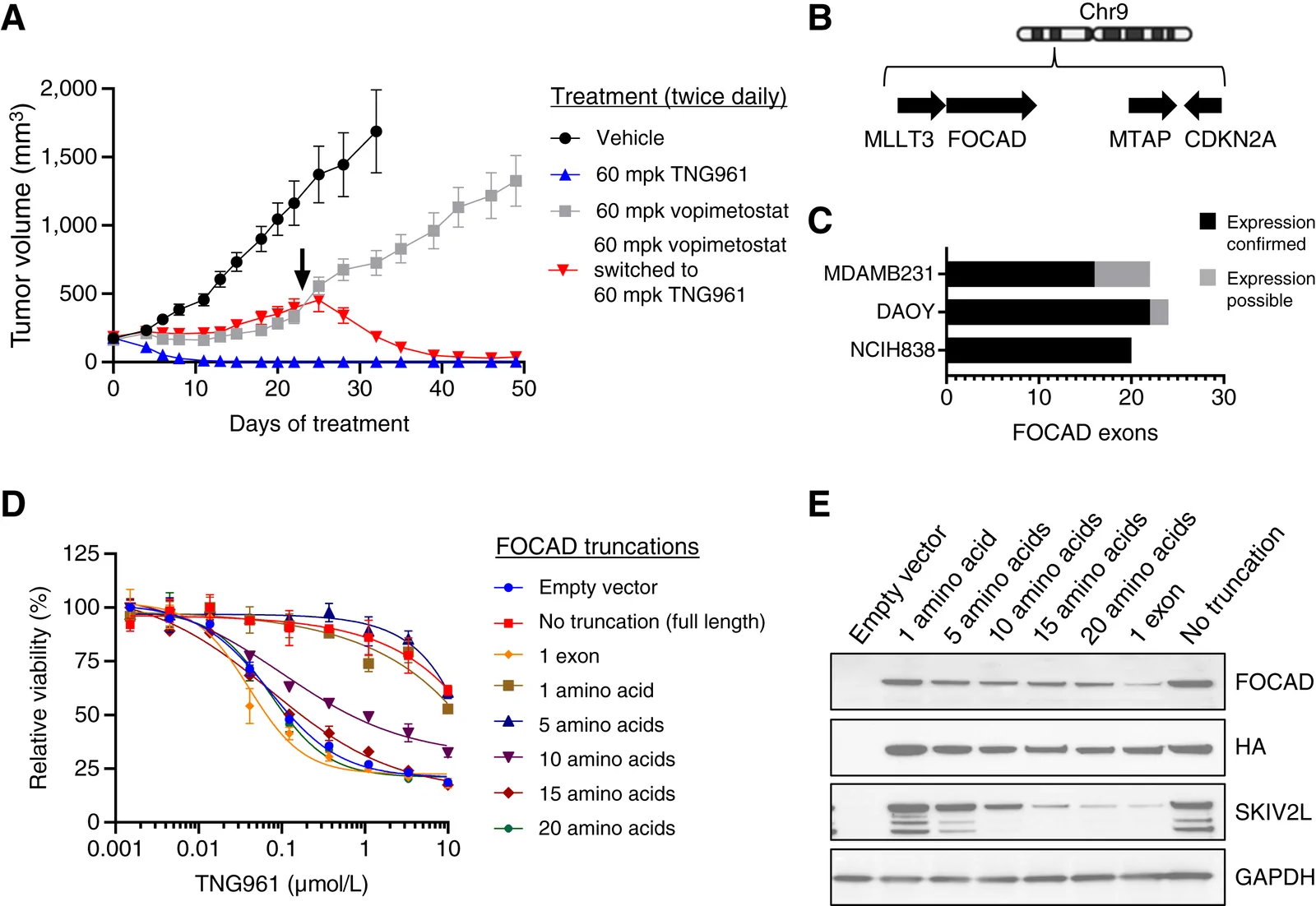
TNG961 shows *in vivo* activity in the PRMT5 inhibitor–refractory setting and inhibits the growth of cells harboring C-terminal *FOCAD* truncations. **A,** Mice bearing MIAPACA2 xenografts were treated orally twice daily with 60 mg/kg vopimetostat (TNG462) or 60 mg/kg TNG961. After 21 days (black arrow), treatment was continued as indicated (blue and gray) or switched from vopimetostat to TNG961 (red). Tumor volumes were measured by calipers (*N* = 8 mice/arm). Data are mean ± SEM. **B,** Schematic showing the tail-to-tail orientation of *FOCAD* and *CDKN2A* on chr9p21. **C,** Endogenous *FOCAD* truncations in MDA-MB-231, DAOY, and NCIH838 cells mapped by qPCR using probes tiled across the *FOCAD* locus. Black segments denote exons with confirmed expression; gray segments denote exons between a positive probe (black) and the first negative probe (end of bar). **D,** NCIH1755 cells were engineered to express HA-tagged *FOCAD* cDNA encoding the indicated C-terminal truncations. Relative viability after 7 days of TNG961 treatment was assessed by CellTiter-Glo. Data are mean ± SD (*N* = 2 biological replicates). **E,** Western blot confirming the expression of HA-tagged FOCAD truncations and the corresponding effects on SKIV2L stability. Representative images are from *N* = 2 biological replicates.

To test this, mice bearing MIAPACA2 xenografts were treated with TNG961 or the MTA-cooperative PRMT5 inhibitor vopimetostat (TNG462; Fig. 7A; ref. 9). TNG961 (60 mg/kg) quickly achieved complete tumor responses, with 8 out of 8 mice showing no measurable tumor within 15 days. In contrast, vopimetostat initially suppressed tumor growth, but tumors progressed after ∼2 weeks, consistent with prior reports for the response of MIAPACA2 tumors using another MTA-cooperative PRMT5 inhibitor (45, 46). In previous studies, we determined that MIAPACA2 tumors that progress on vopimetostat treatment (defined here as > 200% tumor growth from the maximal response observed at day 9) remain MTAP-null and exhibit PRMT5 inhibition, demonstrating that vopimetostat is still pharmacologically active at progression (Supplementary Fig. S6A–S6C). To determine whether tumors progressing on PRMT5 inhibitor treatment remained sensitive to HBS1L degradation, one vopimetostat-treated cohort was switched to TNG961 after 21 days upon evidence of tumor outgrowth (Fig. 7A, black arrow). All tumors in this group rapidly regressed after the compound switch, demonstrating that PRMT5 inhibitors and TNG961 have complementary activity and nonoverlapping resistance mechanisms. All treatments were well tolerated, as indicated by the maintenance of body weight (Supplementary Fig. S6D).

### C-Terminal FOCAD Truncations Phenocopy FOCAD Loss and Confer TNG961 Sensitivity

The *FOCAD* gene is oriented tail-to-tail with *CDKN2A* on chr9p21 (Fig. 7B) such that chromosomal deletions extending from *CDKN2A* can truncate *FOCAD* from the 3′ end. Using qPCR probes tiled across the *FOCAD* locus, we identified multiple cell lines harboring endogenous 3′ truncations in *FOCAD* (Fig. 7C). MDA-MB-231 cells (Fig. 5B) and NCIH838 xenografts (Fig. 5F), both truncated after exon 20, were sensitive to TNG961, indicating functional loss of FOCAD with respect to SKI complex stabilization and consequent HBS1L synthetic lethality.

We next asked whether smaller 3′ truncations could likewise phenocopy complete loss of *FOCAD* and, if so, to define the minimum truncation required for FOCAD inactivation. Using NCIH1755 cells that endogenously lack the entire *FOCAD* gene, we expressed a series of C-terminally truncated FOCAD constructs lacking between 1 and 20 amino acids and assessed TNG961 sensitivity in a 7-day viability assay as a surrogate for FOCAD function (Fig. 7D). Full-length FOCAD (no truncation) and empty vector controls (no FOCAD expression) served as maximal rescue and maximal sensitivity controls, respectively. A truncation of one exon phenocopied complete FOCAD loss. Although truncations of 1 or 5 amino acids retained resistance comparable with full-length FOCAD and a 10-amino-acid truncation showed an intermediate phenotype, truncation of 15 amino acids phenocopied complete FOCAD loss and conferred full sensitivity to TNG961. Thus, removal of as few as 15 C-terminal residues is sufficient to inactivate FOCAD with respect to HBS1L synthetic lethality.

Consistent with our model that FOCAD stabilizes the SKI complex (Fig. 1C), FOCAD truncations that conferred TNG961 sensitivity (15 amino acids, 20 amino acids, or 1 exon) also failed to stabilize the SKI complex component SKIV2L, as assessed by Western blot (Fig. 7E), whereas shorter truncations preserved SKIV2L stability. Together, these data support a model in which the C-terminus of FOCAD is required for the stabilization of the SKI complex and indicate that even tumors harboring small truncations in *FOCAD* may be responsive to TNG961 treatment.

## Discussion

Biomarker-driven clinical development has repeatedly transformed oncology practice. Early successes such as HER2-directed therapy in *ERBB2*-amplified breast cancer helped establish a development template for approvals that was later expanded to multiple genotype-defined settings, including EGFR-, BRAF-, ALK-, KRAS-, and NTRK-driven cancers (47). In parallel, the clinical success of synthetic lethality, most notably with PARP inhibitors in *BRCA1/2*-mutant tumors, validated the broader principle that tumor suppressor gene loss can define actionable vulnerabilities (47). Against this backdrop, chromosome 9p21 loss is among the most common genomic events in human cancer, caused by deletion of the tumor suppressor gene *CDKN2A*, that often extends to collateral co-deletion of nearby genes such as *MTAP* and *FOCAD* (Fig. 1A).

Therapeutically, the chromosome 9p21 deletion context has already motivated the development of synthetic lethal strategies that exploit *MTAP* loss, including clinical efforts with MTA-cooperative PRMT5 inhibitors (e.g., vopimetostat, navlimetostat) and MAT2A inhibitors. In this study, we describe TNG961, an oral, potent, and selective molecular glue degrader of HBS1L developed to exploit an additional vulnerability created by homozygous deletions of chromosome 9p21. *FOCAD* loss occurs in ∼one third of *MTAP*-deleted cancers across diverse histologies (Supplementary Fig. S1A), underscoring a broad therapeutic opportunity. The synthetic lethal relationship between FOCAD loss and the HBS1L–PELO ribosome rescue complex was initially identified through CRISPR-based functional genomics screens (Fig. 1B) and has recently been independently validated by multiple groups (19, 24, 25).

TNG961 is a novel molecular glue degrader and the first pharmacologic agent to exploit HBS1L-FOCAD synthetic lethality, discovered through integrated biochemical and cell-based phenotypic screening (Fig. 2A–C) and subsequent medicinal chemistry efforts (Fig. 2D and E). Mechanistically, TNG961 brings HBS1L and CRBN into proximity, promoting ternary complex formation that facilitates HBS1L ubiquitination followed by target degradation (Fig. 3A–C; Supplementary Fig. S1B–S1E). A cryo-EM structure confirms that TNG961 facilitates the formation of a CRBN–HBS1L ternary complex (Fig. 3D and E) competent for ubiquitination *in vitro* and in cells, which ultimately leads to HBS1L target degradation across diverse cell lines (Fig. 4A). Consistent with this mechanism, TNG961-induced HBS1L degradation and the resulting PELO destabilization are both CRBN- and neddylation-dependent (Fig. 4B and C; Supplementary Fig. S1F). Moreover, TNG961-induced HBS1L degradation is fully rescued by mutation of the conserved glycine residue of the HBS1L degron loop (HBS1L G625N; Fig. 4D), consistent with the analogous G575N mutation in GSPT1 that abrogates CRBN-dependent degradation (31, 37) by the molecular glue degrader MRT-2359 targeting GSPT1 (Supplementary Fig. S1G).

Clinically approved CRBN-based degraders such as thalidomide, lenalidomide, and pomalidomide all carry known teratogenic liabilities linked to the recruitment and degradation of defined neosubstrates, including SALL4 (48). In contrast, TNG961 exhibits proteome-wide selectivity for HBS1L, with no significant effects on other established neosubstrates (Fig. 4E and F; Supplementary Fig. S2A–S2C), with the reduction of PELO being consistent with a secondary destabilizing effect resulting from the loss of its binding partner HBS1L. We observed an apparent dose-dependent increase in CK1α by immunoblot (Fig. 4F) upon TNG961 treatment that was not recapitulated in global proteomics (Fig. 4E; Supplementary Fig. S2A and S2B). The basis for this discrepancy remains unclear and warrants further investigation, but it does not change our overall conclusion that TNG961 is highly selective for HBS1L.

Functionally, TNG961 selectively drives loss of viability in FOCAD-negative, but not FOCAD-positive models, with ∼100-fold selectivity in *FOCAD* isogenic cell line pairs (Fig. 5A and B; Supplementary Fig. S3) and minimal impact on FOCAD-positive cells across a large cancer cell line panel (Fig. 5C). The chromosome 9p21-deletion context was further confirmed in a PRISM screen of >900 cell lines (Supplementary Fig. S4A and S4B). Other genetic alterations that result in the inactivation of the SKI complex, such as microsatellite-driven destabilization of SKI complex member tetratricopeptide repeat domain 37 (TTC37), have also been predicted to show a dependency on HBS1L–PELO function, highlighting the robustness of the interaction and supporting the proposed mechanism (19, 25). Although alternative mechanisms of SKI complex inactivation did not score in our PRISM screen, this may be driven by their low representation or annotation in cell lines.

*In vivo*, TNG961 showed dose-proportional pharmacokinetics and pharmacodynamics, with HBS1L degradation correlating with exposure (Fig. 5D). Robust dose-dependent tumor growth inhibition and regressions were observed across additional FOCAD-negative xenograft models spanning different histologies (Fig. 5E–H; Supplementary Fig. S4C–S4F), supporting FOCAD status as the primary driver of TNG961 sensitivity, rather than tumor lineage.

Mechanistically, HBS1L degradation induces translational arrest in FOCAD-negative, but not FOCAD-reconstituted, cells (Fig. 6A; Supplementary Fig. S5A). Translational arrest and the accumulation of incomplete proteins resultant from stalled ribosomes can trigger the induction of canonical ER stress markers and activation of the UPR, including induction of IFRD1, ATF4, splicing of XBP1, and a well-described downstream transcriptional response (18, 19, 24, 25, 39–44). We observed activation of these canonical ER stress and UPR pathway markers selectively in the FOCAD-negative context *in vitro* (Fig. 6B–E; Supplementary Fig. S5B–S5E), with additional induction of IFRD1 in tumor xenografts (Supplementary Fig. S5C).

As the functional loss of *FOCAD* on chromosome 9p21 invariably co-occurs with *MTAP/CDKN2A* deletion (Fig. 1A), our findings motivate the evaluation of combination strategies with PRMT5 or MAT2A inhibitors. MTA-cooperative PRMT5 inhibitors are clinically active across *MTAP*-deleted tumor types (13, 49); however, maximal benefit for patients will likely be seen using combination therapy. Preclinically, the MIAPACA2 FOCAD/MTAP-null PDAC xenograft model is refractory to MTA-cooperative PRMT5 inhibitors (50, 51), yet TNG961 induces regression in tumors that progress on vopimetostat (Fig. 7A; Supplementary Fig. S6), supporting nonoverlapping sensitivity and resistance mechanisms between these modalities. Collectively, these data suggest that TNG961 may occupy a distinct clinical niche within the broader chr9p21 co-deletion population and that combination or sequencing with MTA-cooperative PRMT5 or MAT2A inhibitors could deepen and/or prolong responses through complementary mechanisms of action.

Though preclinical data support TNG961 monotherapy in FOCAD-null cancers regardless of histology, the mechanism described here (ribosome rescue disruption leading to translation arrest and ER stress/UPR activation) suggests that tumor-specific combinations with selected cytotoxic or targeted backbones may be attractive due to differentiated mechanisms of action. Clinically, however, the standard of care (SOC) varies sharply by histology and line of therapy, making pan-histology combination strategies difficult to standardize. Accordingly, we believe TNG961 is best advanced as monotherapy in *FOCAD*-deleted tumors in a histology-agnostic fashion. Once single-agent activity is established, a clinical development path would take either of two paths: (i) continued histology-agnostic monotherapy expansion and/or (ii) focused development in tumor-specific cohorts (such as NSCLC) that evaluate a small, prespecified set of SOC backbones in earlier lines in which regimens are more defined. This monotherapy-first strategy is supported by the broad distribution of *FOCAD* deletion (Supplementary Fig. S1A) and by FOCAD status, rather than lineage, driving TNG961 sensitivity across *in vitro* and *in vivo* models (Fig. 5).

In terms of FOCAD-negative patient selection, we show that functional loss of FOCAD, either through complete deletion or small C-terminal truncations, renders cells sensitive to TNG961 (Fig. 7B–D) and destabilizes the SKI complex component SKIV2L (Fig. 7E), providing a mechanistic link between the genetic context of *FOCAD* loss and the molecular target HBS1L. Notably, truncation of as few as ∼15 to 20 C-terminal residues functionally phenocopies complete FOCAD protein loss (Fig. 7D). This observation has practical implications for prospective patient-selection assay design, including the choice of antibody epitopes to minimize false-negative classification and for the interpretation of DNA-based sequencing approaches with exon-level breakpoint resolution.

Collectively, the present study establishes TNG961 as an oral, potent, and selective first-in-class degrader targeting the HBS1L/PELO ribosome rescue complex, validates FOCAD loss as a clinically actionable biomarker, and supports clinical development strategies that may include histology-agnostic, biomarker-defined monotherapy with staged, indication-tailored combinations where feasible.

## Methods

Key reagents (Supplementary Table S1); cell lines and culture conditions (Supplementary Table S2); cDNA, sgRNA, and other DNA/RNA sequences (Supplementary Table S3); and cryo-EM data collection, refinement, and validation statistics (Supplementary Table S4) are available in Supplementary Materials.

### Chemical Compounds

MG132 was purchased from Selleck Chemicals (S2619). MLN4924 was purchased from MedChemExpress (HY-70062). CC-885 was purchased from MedChemExpress (HY-101488). TNG-6266 was purchased from Enamine (Z5000160111). TNG961 was synthesized at WuXi AppTec (lots WX-TJ-HC-1120-01 and WX-TJ-HC-1120-02; average purity 99.1%).

### Protein Expression and Purification

#### Full-Length Human HBS1L (for Biochemical Assays)

The expression construct contained an N-terminal 6×His tag, a TEV protease cleavage site, and an Avi tag (His-TEV-Avi-HBS1L) and was cloned into pFastBac1 (Invitrogen 10359016) and expressed in Sf9 cells. *In vivo* biotinylation was achieved by coexpressing BirA with HBS1L in insect cells. Following 48 hours of expression at 27°C, cells were harvested and resuspended in Buffer A [50 mmol/L Tris, pH 8.0; 500 mmol/L NaCl, 10% (v/v) glycerol; 1 mmol/L Tris (2-carboxyethyl)-phosphin-HCl (TCEP)] supplemented with 10 mmol/L imidazole, benzonase (125 U/mL cell culture), and protease inhibitor cocktail (Roche, 05056489001). Cells were lysed by sonication (3 seconds on/3 seconds off at 200 W). Lysate was clarified twice by centrifugation (30 minutes at 20,300 × *g*), and the supernatant was incubated with 5 mL of pre-equilibrated Ni-NTA resin (QIAGEN, 30230) for 1 hour at 4°C. The resin was loaded into a column, washed, and the protein was eluted with Buffer A containing stepwise increases in imidazole (20, 50, 250 mmol/L). Eluted protein was pooled and incubated overnight at 4°C with His-tagged TEV protease (1:2 w/w). Samples were reapplied to Ni-NTA to remove TEV protease and undigested protein. The flow-through was loaded onto a Strep-Tactin 4Flow column (IBA Lifesciences, 2-1250-010) equilibrated in Buffer A, washed, and eluted with Buffer A supplemented with 50 mmol/L biotin. Pooled fractions were concentrated and further purified by size exclusion chromatography (SEC) on a Superdex 200 10/300 column (Cytiva, 28990944) in SEC buffer (10 mmol/L HEPES, pH 7.0; 240 mmol/L NaCl; 3 mmol/L TCEP). Purified protein (3.3 mg/mL) was aliquoted and stored at −80°C.

#### Truncated Human HBS1L (Used for Structural Studies)

The construct encoded an N-terminal 6×His-tagged maltose binding protein (MBP) followed by a TEV protease cleavage site and HBS1L (478–684; His-MBP-TEV-HBS1L) and was expressed in Hi-5 insect cells using baculovirus for 48 hours at 27°C. Lysis and purification through TEV cleavage were performed as described for full-length HBS1L. After the second Ni-NTA step, flow-through was applied to a Resource S column (Cytiva, 17118001) equilibrated in Buffer A containing 250 mmol/L NaCl, and protein was eluted using a linear gradient to 1.0 mol/L NaCl. Pooled fractions were concentrated and purified by SEC on a Superdex 75 10/300 column (GE HealthCare) in final buffer (10 mmol/L HEPES, pH 8.0; 240 mmol/L NaCl, 3 mmol/L TCEP; 5% glycerol). Purified protein (8.4 mg/mL) was aliquoted and stored at −80°C.

#### Full-Length Human GSPT1 (Used for Biochemical Assays)

This construct was cloned into pET21a and expressed in *Escherichia coli* BL21-Gold(DE3). The construct contained an N-terminal 6×His tag, SUMO tag, and Avi tag (His-SUMO-Avi-GSPT1). Expression was induced with 0.2 mmol/L IPTG at OD_600_ = 0.6 for 12 hours at 16°C. Cells were harvested and resuspended in Buffer A supplemented with 10 mmol/L imidazole, 1 mmol/L phenylmethylsulfonyl fluoride (PMSF), and 25 U/mL benzonase. Cells were lysed by sonication and clarified by centrifugation (30 minutes at 20,300 × *g*). The supernatant was incubated with 10 mL pre-equilibrated Ni-NTA (QIAGEN, 30230) for 1 hour at 4°C, washed, and eluted with Buffer A using stepwise imidazole (20, 50, 250 mmol/L). The eluted protein was pooled and incubated overnight at 4°C with Ulp1 protease (1:100 w/w) while dialyzing into 50 mmol/L Tris, pH 8.0; 75 mmol/L NaCl; 7.5 mmol/L MgCl_2_; 1 mmol/L TCEP. The digested protein was then biotinylated by adding His-BirA (1:150 w/w), plus 0.5 mmol/L biotin and 1 mmol/L ATP, and incubating for 4 hours at 18°C. The sample was passed over Ni-NTA to remove His-BirA and undigested protein. The flow-through was loaded onto a HiTrap Heparin HP column (Cytiva, 17040601) and eluted with a linear gradient from 50 mmol/L to 1.0 mol/L NaCl in buffer containing 50 mmol/L Tris, pH 8.0; 10% glycerol; and 1 mmol/L TCEP. Fractions were pooled, concentrated, and polished by SEC using a Superdex 200 10/300 column in 10 mmol/L HEPES, pH 7.0; 240 mmol/L NaCl; and 3 mmol/L TCEP. Purified protein (4.9 mg/mL) was aliquoted and stored at −80°C.

#### CRBN–DDB1 Complexes

For ternary complex formation assays, CUL4A–RBX1–DDB1–CRBN was obtained commercially (R&D Systems, E3-650 or ChemPartner 202401264101). For cryo-EM studies, CRBN (40–442) with full-length DDB1 was produced in insect cells as previously described, except that a TEV cleavage site replaced the thrombin cleavage site in the CRBN construct. Initial Ni-NTA purification was performed as for full-length HBS1L; CRBN–DDB1 eluted in 20 and 50 mmol/L imidazole fractions. Pooled fractions were incubated overnight at 4°C with TEV protease (1:10 w/w) and reapplied to Ni-NTA to remove TEV protease and undigested protein. CRBN–DDB1 from the flow-through and 20 mmol/L imidazole wash was diluted to 50 mmol/L NaCl and applied to a HiTrap Heparin HP column (Cytiva, 17040601); contaminants bound to the resin, and CRBN–DDB1 was collected in the flow-through. The complex was further purified by SEC on a Superdex 200 26/600 column (Cytiva, 28989336) in 10 mmol/L HEPES, pH 7.0; 240 mmol/L NaCl; and 3 mmol/L TCEP. Purified protein was concentrated to >16 mg/mL, aliquoted, and stored at −80°C.

### Cryo-EM

#### Complex Assembly and Grid Preparation

The HBS1L–CRBN–DDB1 + TNG-4857 and TNG961 complexes were assembled by copurification prior to grid preparation. HBS1L (478–684) and CRBN (40–442)/DDB1 were mixed 1:1 (molar) with a 3.3-fold molar excess of glue compound in 10 mmol/L HEPES, pH 7.0; 250 mmol/L NaCl; and 3 mmol/L TCEP, incubated overnight at 4°C, and purified by SEC on Superdex 200 10/300. A single SEC peak contained all three proteins, as confirmed by SDS-PAGE. The purified complex (8 mg/mL) was mixed with 0.2% n-octylglucoside and immediately used for grid preparation. The sample was applied to glow-discharged UltrAuFoil gold grids (R1.2/1.3, 300 mesh; Electron Microscopy Sciences, Q350AR13A), blotted for 2 seconds, and vitrified in liquid ethane using a Vitrobot IV (Thermo Fisher Scientific, RRID:SCR_025773) at 8°C and 100% humidity.

#### Data Acquisition and Processing

Data were collected on a 300 kV Titan Krios G4 (Thermo Fisher Scientific) equipped with a Falcon 4i detector operated in counting mode (Viva Biotech). Movies were recorded at 130,000× magnification (pixel size 0.932 Å), with a total dose of 48 e^−^/Å^2^ fractionated into 40 frames. A total of 16,029 and 9,532 movies were collected for TNG-4857 and TNG961, respectively, with a defocus range of −1.0 to −2.2 μm, using a Selectris X energy filter (20 eV slit width). Processing was performed in RELION v5.0 (RRID:SCR_016273) with motion correction and contrast transfer function (Contrast Transfer Function (CTF) estimation followed by auto-picking and particle extraction. For TNG961, approximately 4.5 mol/L particles were carried forward to 2D classification, *ab initio* reconstruction, and 3D classification. A set of 898,000 particles from the best class was subjected to additional 3D classification, CTF refinement, and 3D refinement, yielding 669,000 particles used to reconstruct a map at 2.9 Å global resolution. For TNG-4857, approximately 10 mol/L initial particles were refined via the same process, resulting in 661,000 particles that were used to construct the final map. Initial models were generated by rigid-body docking of CRBN/DDB1 (PDB 5HXB) and HBS1L (unpublished), followed by iterative refinement in Coot (RRID:SCR_014222) and Phenix (RRID:SCR_014224). Figures were generated in PyMOL (Schrödinger, RRID:SCR_000305). Cryo-EM data collection, refinement, and validation statistics are reported in Supplementary Table S4.

### Cell Line Engineering and Cell Culture

#### Cell Culture

Cells were maintained at 37°C in a humidified incubator with 5% CO_2_. Mycoplasma testing was performed weekly using MycoAlert (Lonza, LT07-318). Cell lines were authenticated by short tandem repeat profiling (LabCorp) and used at ≤15 passages and within 8 weeks of thawing. Cell line sources, culture conditions, plasmid insert sequences, and key reagents are listed in Supplementary Tables S1–S3.

#### Lentiviral Transduction

For engineering stable cell lines, lentivirus was produced by transfecting Lenti-X cells (Takara Bio, 632180) overnight with the plasmid of interest and packaging mix (Cellecta, CPCP-K2A) using Lipofectamine 3000 (Thermo Fisher Scientific, L3000015) in Opti-MEM (Gibco, 31985-062). After 16 to 24 hours, the media was replaced with fresh DMEM + 30% FBS. Viral supernatant was harvested 48 hours after transfection and filtered (0.45 μm). Target cells were infected overnight with virus-containing media supplemented with 8 μg/mL polybrene (Sigma-Aldrich, TR-1003). Cells recovered for 24 hours prior to antibiotic selection based on the construct resistance cassette using blasticidin (Gibco, A1113903), puromycin (Gibco, A1113803), or geneticin (Gibco, 10-131-035).

#### HiBiT CRISPR Knock-In Cell Line

HBS1L-HiBiT cells were generated by knocking HiBiT into the endogenous HBS1L locus of HEK293 (LgBiT) cells (Promega, N2672) using a Ribonucleoprotein (RNP)-based) CRISPR editing workflow. A 24 µmol/L tracrRNA:crRNA duplex was prepared from 100 µmol/L Alt-R Cas9 crRNA and 100 µmol/L Alt-R Cas9 tracrRNA in duplex buffer, heated to 95°C for 5 minutes, and cooled to room temperature. TruCut Cas9 (20 µmol/L) was added and incubated for 10 to 20 minutes to form RNPs; the final RNP concentration for nucleofection was 4 µmol/L. Cells were lifted with a nonenzymatic reagent (Sigma, C5914), pelleted (90 × *g*, 10 minutes, room temperature), and resuspended in SF buffer (Lonza, V4XC-2032). Each nucleofection used 2 × 10^6^ cells, 4 µmol/L RNP, 100 µmol/L donor template (IDT), and 100 µmol/L electroporation enhancer (IDT, 1075915), delivered in 25 μL using a 4D-Nucleofector X Unit (Lonza, AAF-1003X; RRID SCR_023155; program CM-130; two pulses). Cells were recovered in prewarmed media (80 μL), transferred to six-well plates, and cultured with penicillin-streptomycin.

To assess HiBiT expression, 1 × 10^4^ cells in 40 μL were seeded in triplicate into white 384-well plates together with HEK293 GSPT1-HiBiT–positive control cells (Promega, CS3023106); media-only wells served as background. After 15 minutes of equilibration, 2× lytic reagent (Promega, N3040; prepared by mixing 20 μL substrate per 1 mL lytic buffer) was added (40 μL/well), and plates were shaken for 20 minutes at room temperature. Luminescence was measured on PHERAstar (RRID SCR_027001) or EnVision (RRID SCR_018038) plate readers (PHERAstar: gain 3600, 0.1 seconds integration; EnVision: 0.5 seconds integration). Single-cell clones were isolated from positive pools, and correct HiBiT integration was confirmed by PCR and sequencing.

### HiBiT Assays

#### Endpoint (Lytic) HiBiT Assay

Cells were seeded at 10,000 cells per well in 384-well plates in DMEM with 10% FBS and allowed to adhere overnight. Compounds were then added and incubated for 24 hours, after which detection of HiBiT-tagged proteins was performed using the Nano-Glo HiBiT Lytic Detection System (Promega, N3040) according to the manufacturer’s instructions. Luminescence was measured using an EnVision plate reader (Revvity, RRID SCR_018038) with a 0.1-second integration time.

#### Live-Cell Kinetic HiBiT Assay

Cells were seeded at 20,000 cells per well in 384-well plates in CO_2_-independent medium (Invitrogen, 18045088) supplemented with 1 mmol/L sodium pyruvate (Thermo Fisher Scientific, 11360070), 2 mmol/L L-glutamine (Thermo Fisher Scientific, 25030081), and 1× penicillin-streptomycin (Thermo Fisher Scientific, 15140163) and allowed to adhere overnight. The following day, Nano-Glo Endurazine Live Cell Substrate (Promega, N2570) was added according to the manufacturer’s instructions and incubated for 2 hours. Next, treatments were added, and luminescence was measured immediately with a PHERAstar plate reader (BMG, RRID: SCR_027001) at indicated time intervals.

### TR-FRET Ternary Complex Formation Assay

TR-FRET proximity assays to quantify compound-induced ternary complex formation were performed at ChemPartner (China). CC-885 and TNG961 were dispensed (top concentration 10 µmol/L final; 10-point, 3-fold serial dilution) into PerkinElmer OptiPlate-384 using an Echo550 acoustic dispenser (Labcyte, RRID:SCR_027476). Reactions were assembled in 1× assay buffer (50 mmol/L HEPES, pH 8.0; 50 mmol/L NaCl; 5 mmol/L MgCl_2_; 0.01% BSA; 0.05% Tween-20; 1 mmol/L TCEP) by adding 5 μL of 2× His-tagged CUL4A–RBX1–DDB1–CRBN complex (25 nmol/L for GSPT1 reactions or 50 nmol/L for HBS1L reactions; ChemPartner, 20240126410), followed by 5 μL of 2× Avi-tagged protein (50 nmol/L HBS1L or 25 nmol/L GSPT1; Viva IDs as listed). After a 1-hour incubation at room temperature, 10 µL of 2× detection mix (0.67 nmol/L anti–6×His-Tb cryptate Gold, Revvity, 61HI2TLB; 83.33 nmol/L streptavidin-XL665, Revvity, 610SAXLB) was added. Plates were centrifuged (1,000 rpm, 30 seconds) and incubated for 3 hours at 25°C. TR-FRET was read on an EnVision plate reader (Revvity; excitation 340 nm; emissions 615/665 nm RRID:SCR_018038). Signal was calculated as the 665/615 ratio and converted to %Activation using %Activation = 100 × (Ratio *−* Min)/(Max − Min), where Max was defined by 10 µmol/L CC-885 and Min was defined by DMSO-only wells. Dose–response curves were fit in XL-Fit (model 205) to determine AC_50_ values. B_max_ denotes the maximal ternary complex signal relative to the assay control.

### Hit Finding (Parallel Screening)

A proprietary CRBN-focused molecular glue library was assembled through custom synthesis and commercial purchase (Enamine) and formatted as 10 mmol/L DMSO stocks in 384-well plates. The small-molecule library comprised ∼3,500 drug-like compounds (average MW = 429 g/mol) with CRBN-targeting (e.g., glutarimides) and other E3-targeting warheads (e.g., DCAF15). The library was screened in parallel using (i) the HiBiT endpoint degradation assay in HBS1L-HiBiT and GSPT1-HiBiT engineered cells (10 µmol/L; 24 hours; final volume 40 μL; 1.25% DMSO) and (ii) the TR-FRET ternary complex assay (screening format: 1 µmol/L; 1% DMSO; 20 μL volume; HBS1L 25 nmol/L, GSPT1 12.5 nmol/L; 0.33 nmol/L Mab Anti–6HIS-Tb cryptate Gold and 41.67 nmol/L streptavidin-XL665 detection mix). For the plate layout (Supplementary Fig. S7A and S7B), CC-885 and CC-90009 were included as GSPT1 degrader controls for the cellular screen; CC-885 served as a positive control for the ternary complex screen. Cellular and ternary complex screens were performed in duplicate (*N* = 2), with statistical performance and correlation between replicate plates calculated (Supplementary Fig. S7C–S7E). Hits meeting criteria (degradation >20% with <20% viability loss) were confirmed in single-point and dose-response HiBiT assays and further validated by sensitivity to proteasome inhibition (MG132) and NEDD8-activating enzyme inhibition (MLN4924, pevonedistat). Hits from the ternary complex screen were confirmed in single-point and dose-response TR-FRET assays.

### *In Vitro* Ubiquitination Assay

Molecular glue–mediated ubiquitination reactions (20 μL) contained 25 nmol/L UBA1 (South Bay Bio, SBB-CE0011), 25 nmol/L UBE2D1 (South Bay Bio, SBB-CE0021), 20 nmol/L CUL4A–RBX1–DDB1–CRBN (ChemPartner, 20240126410), 1 µmol/L ubiquitin (LifeSensors, SI-0201-5000), 100 µmol/L ATP (Thermo Fisher Scientific, R0441), and 20 nmol/L HBS1L or GSPT1 (Viva, IDs as listed above), plus 10 µmol/L CC-885 (positive control) or TNG961, in assay buffer (ChemPartner). Negative controls omitted ATP or the protein of interest. Reactions were incubated for 3 or 24 hours at 25°C and quenched with 4× lithium dodecyl sulfate (LDS) sample buffer. Samples (10 ng of the protein of interest per lane) were resolved on NuPAGE Novex 4% to 12% Bis-Tris gels and transferred to 0.2 μm nitrocellulose membranes (iBlot). Membranes were blocked in Odyssey blocking buffer (LICORbio, 927-50000) for 1 hour and incubated overnight at 4°C with primary antibodies (antiubiquitin P4D1, CST, #3936; anti-HBS1L, Proteintech, 10359-1-AP; anti-GSPT1, CST, 14980S; all 1:1,000). After TBST washes (50 mmol/L Tris, pH 7.0; 150 mmol/L NaCl; 0.05% Tween-20), membranes were incubated for 1 hour at room temperature with LICOR secondaries (anti-mouse 926-68072 or anti-rabbit 926-32213; 1:10,000), washed, and imaged on an Odyssey system (scan setting 169 μm, medium quality RRID:SCR_023227).

### Immunoblotting

#### Western Blot

Cells were rinsed once with ice-cold 1× PBS, and lysis was directly performed using RIPA buffer (Life Technologies, 89901) supplemented with protease and phosphatase inhibitors (Life Technologies, 78445). Lysates were clarified by centrifugation at 20,000 × *g* for 10 minutes. Protein concentration was quantified using a BCA Protein Assay (Thermo Fisher Scientific, 23227) or Pierce 660 nmol/L Protein Assay (Thermo Fisher Scientific, 22660). An equal volume of LDS sample buffer containing dithiothreitol (DTT; Thermo Fisher Scientific, B0008/B0009) was added to the lysate, and samples were heated at 95°C for 5 to 10 minutes. Proteins were resolved via electrophoresis on 4% to 12% Bis-Tris gels (Thermo Fisher Scientific, WG1402BOX; 20–40 μg/lane) and transferred to nitrocellulose membranes (Thermo Fisher Scientific, IB33002).

#### Simple Western (Jess)

Cell pellets were lysed in RIPA buffer (Thermo Fisher Scientific, 89900) supplemented with protease inhibitors (Thermo Fisher Scientific, 1861281) and universal nuclease (VWR, PI88701). Lysates were clarified (10,000 × *g*, 10 minutes, 4°C), and protein concentration was measured using Pierce Rapid Gold BCA (Thermo Fisher Scientific, A53225). Simple Western analysis was performed on Jess (Bio-Techne, RRID:SCR_025095) per the manufacturer’s protocol and analyzed using Compass software v6.2.0. Antibody sources and dilutions are listed in Supplementary Table S1.

### Global Proteomics

Cells (MM.1s) were treated as indicated, and pellets were snap-frozen and stored at −80°C. Frozen pellets were ground under liquid nitrogen and lysed in 1% SDS with 1% protease inhibitor (4× sample volume), followed by sonication. Lysates were clarified (12,000 × *g*, 10 minutes, 4°C), and protein concentration was determined by BCA. Protein was acetone-precipitated (1 volume cold acetone mixed, then 4 volumes cold acetone; −20°C for 2 hours), centrifuged (4,500 × *g*, 5 minutes), washed 2 to 3 times in cold acetone, and dried. Pellets were resuspended in triethylammonium bicarbonate (TEAB) (200 mmol/L final), sonicated, and digested with trypsin (1:50, w/w) overnight. Samples were reduced with DTT (5 mmol/L; 56°C, 30 minutes) and alkylated with iodoacetamide (11 mmol/L; 15 minutes, dark, room temperature).

Peptides were dissolved in solvent A (0.1% formic acid, 2% acetonitrile in water) and loaded onto a reversed-phase analytic column (25 cm, 100 μm i.d.). Peptides were separated on a NanoElute UHPLC (Bruker, RRID:SCR_026972) using solvent A and solvent B (0.1% formic acid in acetonitrile) with the following gradient at 450 nL/minute: 0 to 70 minutes, 6% to 24% B; 70 to 84 minutes, 24% to 35% B; 84 to 87 minutes, 35% to 80% B; 87 to 90 minutes, 80% B. Data were acquired on a timsTOF Pro (RRID SCR_026544) in dia-PASEF mode (full MS/MS scan range 100–1,700; 10 PASEF MS/MS scans per cycle).

### Cell Viability (CellTiter-Glo)

Cells were seeded in 96-well plates to achieve ∼80% confluency at endpoint and allowed to adhere overnight and were treated the following day. After 7 days of compound treatment, ATP levels were assessed as a surrogate for cell viability using CellTiter-Glo (Promega, G9241) according to the manufacturer’s instructions. Luminescence was measured using an EnVision plate reader (Revvity, SCR_018038).

### Xenograft Studies

*In vivo* studies were conducted at Pharmaron (China) under Institutional Animal Care and Use Committee–approved protocols and Association for Assessment and Accreditation of Laboratory Animal Care International guidelines. Female 6- to 8-week-old mice were inoculated subcutaneously in the flank as follows: MIAPACA2, 1 × 10^7^ cells in BALB/c nude mice (Jiangsu JCYK Bioscience Co.), treatment start ∼150 to 200 mm^3^; NCIH838, 1 × 10^7^ cells in BALB/c nude mice (JCYK), start ∼150 mm^3^; RS411, 1 × 10^7^ cells in NOD SCID mice (AKYB), start ∼100 to 150 mm^3^; and NCIH1755, 1 × 10^7^ cells in NOD SCID mice (AKYB), start ∼100 to 150 mm^3^. TNG961 was formulated in 10% DMSO/70% PEG400/20% water, and vopimetostat was formulated in 5% DMA/20% Captisol in water. Tumors were measured twice weekly using calipers, and volumes were calculated as (length × width × width)/2. Body weights were monitored daily for dosing and recorded twice weekly. Where indicated, blood was collected for pharmacokinetics, and tumors were harvested for pharmacodynamics.

### Puromycin Incorporation Assay

Cells were seeded to reach 70% to 80% confluence at the assay end. After treatment, media was removed, and cells were washed with PBS (Invitrogen, 10010001). Puromycin (20 µmol/L; catalog as listed in Supplementary Table S1) was added in prewarmed media and incubated for 15 minutes at 37°C. Cells were washed with ice-cold PBS and lysed directly in plates using ice-cold RIPA buffer (Thermo Fisher Scientific, 89900) supplemented with Halt protease/phosphatase inhibitor (Thermo Fisher Scientific, 78446). Equal amounts of protein were resolved on 4% to 12% Bis-Tris gels (Thermo Fisher Scientific, WG1403), and puromycin-labeled nascent peptides were detected using an anti-puromycin antibody (Sigma-Aldrich, MABE343). Puromycin signal was normalized to Ponceau staining (Thermo Fisher Scientific, A40000279).

### qPCR

Cells were washed with DPBS and lysed in RNA Cell Lysis Buffer (Boston Bioproducts, R-108) supplemented with RNasin (Promega, N2515; 1:40). TaqMan Fast Virus 1-Step Master Mix (Life Technologies, 4444436) and probes listed in Supplementary Table S1 were used per the manufacturer’s instructions. RPLP0 served as the reference gene. Relative mRNA levels were calculated using the ΔΔCt method compared with DMSO-treated controls.

### Data Analysis (GraphPad PRISM, ImageJ, and Spotfire for Visualizations)

Data analysis was performed using GraphPad Prism (GraphPad Software, RRID SCR_002798) and Spotfire (TIBCO Software, RRID SCR_008858). Densitometry for conventional Western blots was performed in ImageJ (NIH, RRID SCR_003070).

## Supplementary Material

Table S1 Key reagents

Table S2 Cell lines and culture conditions

Table S3 cDNA, sgRNA, and other DNA/RNA sequences

Table S4 Cryo-EM data collection

Supplementary DataSet 1 Proteomics

Figure S1 TNG961 is a CRBN-dependent HBS1L molecular glue degrader

Figure S2 TNG961 has proteome-wide selectivity for HBS1L

Figure S3 TNG961 selectively inhibits growth of FOCAD-negative cells

Figure S4 TNG961 selectivity in PRISM cell line panel

Figure S5 Translational suppression and ER stress/UPR activation

Figure S6 TNG961 activity in PRMT5 inhibitor-refractory tumors

Figure S7 Plate layout and performance of primary HBS1L screens

## Data Availability

The data that support the findings of this study are available by request from the corresponding author. The atomic coordinates for the structures of TNG-4857 and TNG961 bound to the HBS1L–CRBN–DDB1 complex have been deposited in the PDB with accession codes 11MR and 10AY, respectively. The associated cryo-EM maps are available in the EMDB with accession codes EMD-75840 and EMD-75038.
