# Supplementary material for: TNG961 Is a Selective Oral HBS1L Molecular Glue Degrader for the Treatment of FOCAD-Deleted Cancers
Source: Cancer Discov. 2026 Apr 19;16(9):1884–902. doi: 10.1158/2159-8290.CD-26-0040 (PMC13531002; doi:10.1158/2159-8290.CD-26-0040)
Supplement: Supplementary file 1 [file cd-26-0040_figure_s1_suppsf1.pdf]

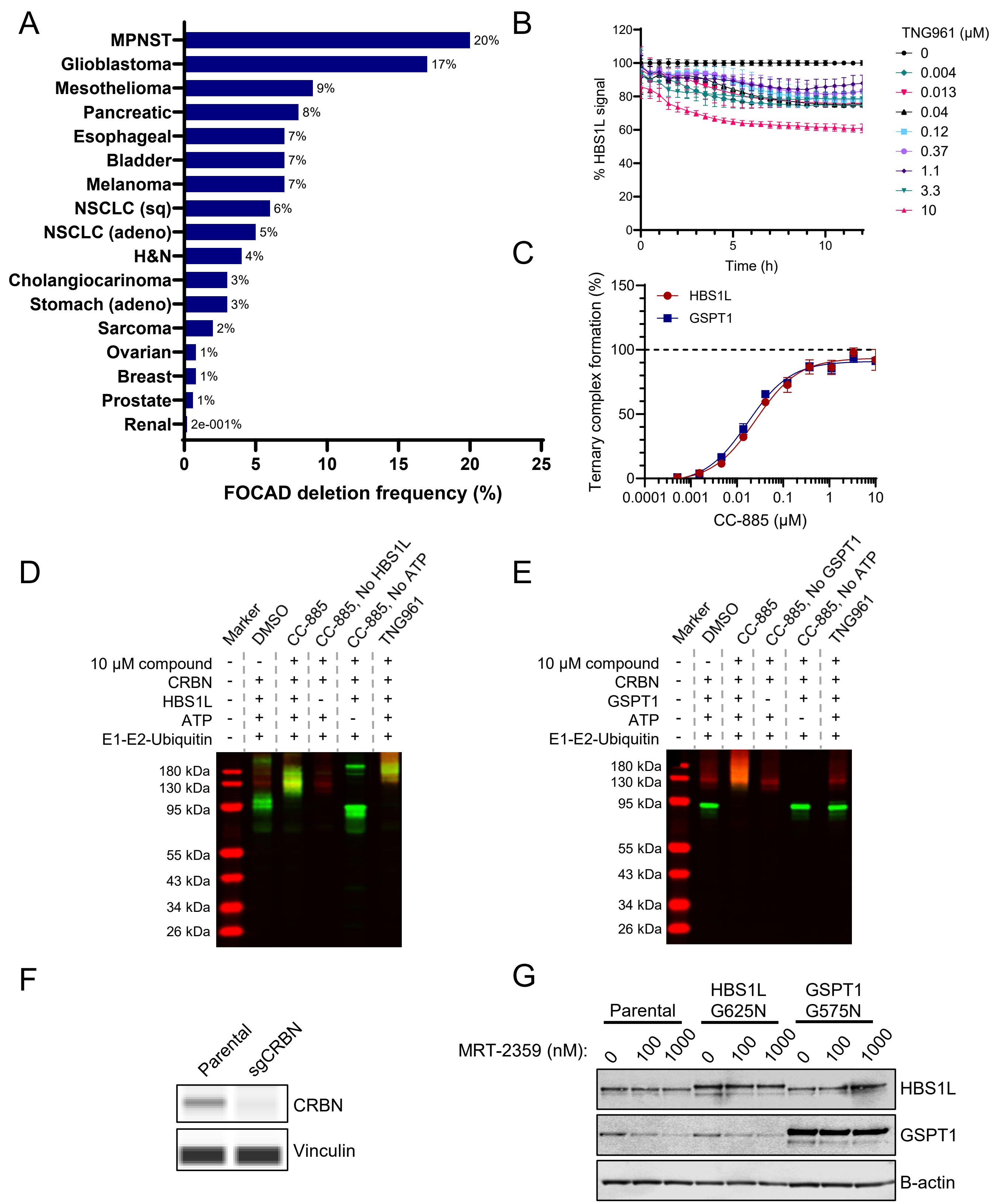

Supplementary Figure S1. FOCAD deletion is prevalent across tumor histologies, and TNG961 is a selective, CRBN-dependent HBS1L molecular glue degrader distinct from non-selective GSPT1 glues**.**

Legend continues on next page

Supplementary Figure S1. FOCAD deletion is prevalent across tumor histologies, and TNG961 is a selective, CRBN-dependent HBS1L molecular glue degrader distinct from non-selective GSPT1 glues**. A,** FOCAD deletion frequency across tumor types (TCGA Pan-Cancer Atlas). MPNST, malignant peripheral nerve sheath tumor; NSCLC, non-small cell lung cancer; sq, squamous; adeno, adenocarcinoma; H&N, head and neck. **B,** Live-cell kinetic degradation counter-assay in 293T GSPT1-HiBiT knock-in cells treated with the indicated concentrations of TNG961. HiBiT signal (GSPT1 protein abundance) was measured every 30 min for 12 h. Data are mean ± SD from N=2 biological replicates. **C,** Dose-dependent TR-FRET ternary complex formation induced by the non-selective molecular glue CC-885 between Terbium-labelled anti-His bound Cul4A–Rbx1–DDB1–CRBN and Streptavidin conjugated XL665 bound biotinylated Avi-tagged HBS1L (red) or biotinylated Avi-tagged GSPT1 (blue) across a CC-885 dose range. Data are mean ± SD from N=3 independent experiments, each with 2 technical replicates. Dotted line indicates CC-885 maximal ternary complex signal (normalized to 100% for comparison). **D, E,** Cell-free ubiquitylation assay using recombinant HBS1L (**D**) or GSPT1 (**E**) in the presence of ubiquitylation machinery (E1/E2/ubiquitin/E3) and ATP. CC-885 (10 μM) is a positive control for both proteins; lanes 2, 4 and 5 serve as no-ubiquitylation controls. TNG961 promotes ubiquitylation (red signal with upward shift in molecular weight in lane 6) of HBS1L (**D**), but not GSPT1 (**E**) (green band does not shift in molecular weight in lane 6). Recombinant HBS1L (**D**) and GSPT1 (**E**) in the presence of 10 μM CC-885 display an upward molecular weight shift compared to the TNG961 lane, consistent with TNG961 being a selective HBS1L molecular glue. Representative image of N=2 independent experiments. **F**, Immunoblot confirming CRBN knockout (sgCRBN) and vinculin as loading control in cells used in CRBN-dependence experiment (related to main **Figure 4B**). Representative image of N=2 independent experiments. **G**, Immunoblot of HBS1L and GSPT1 after 24 h treatment with the GSPT1 molecular glue degrader MRT-2359 in parental MIAPACA2 cells, or in engineered MIAPACA2 lines expressing HBS1L G625N cDNA (covering endogenous HBS1L knockout) or GSPT1 G575N cDNA (covering endogenous GSPT1 knockout). Representative images of N=2 independent experiments.
