# Supplementary material for: TNG961 Is a Selective Oral HBS1L Molecular Glue Degrader for the Treatment of FOCAD-Deleted Cancers
Source: Cancer Discov. 2026 Apr 19;16(9):1884–902. doi: 10.1158/2159-8290.CD-26-0040 (PMC13531002; doi:10.1158/2159-8290.CD-26-0040)
Supplement: Supplementary file 2 [file cd-26-0040_figure_s2_suppsf2.pdf]

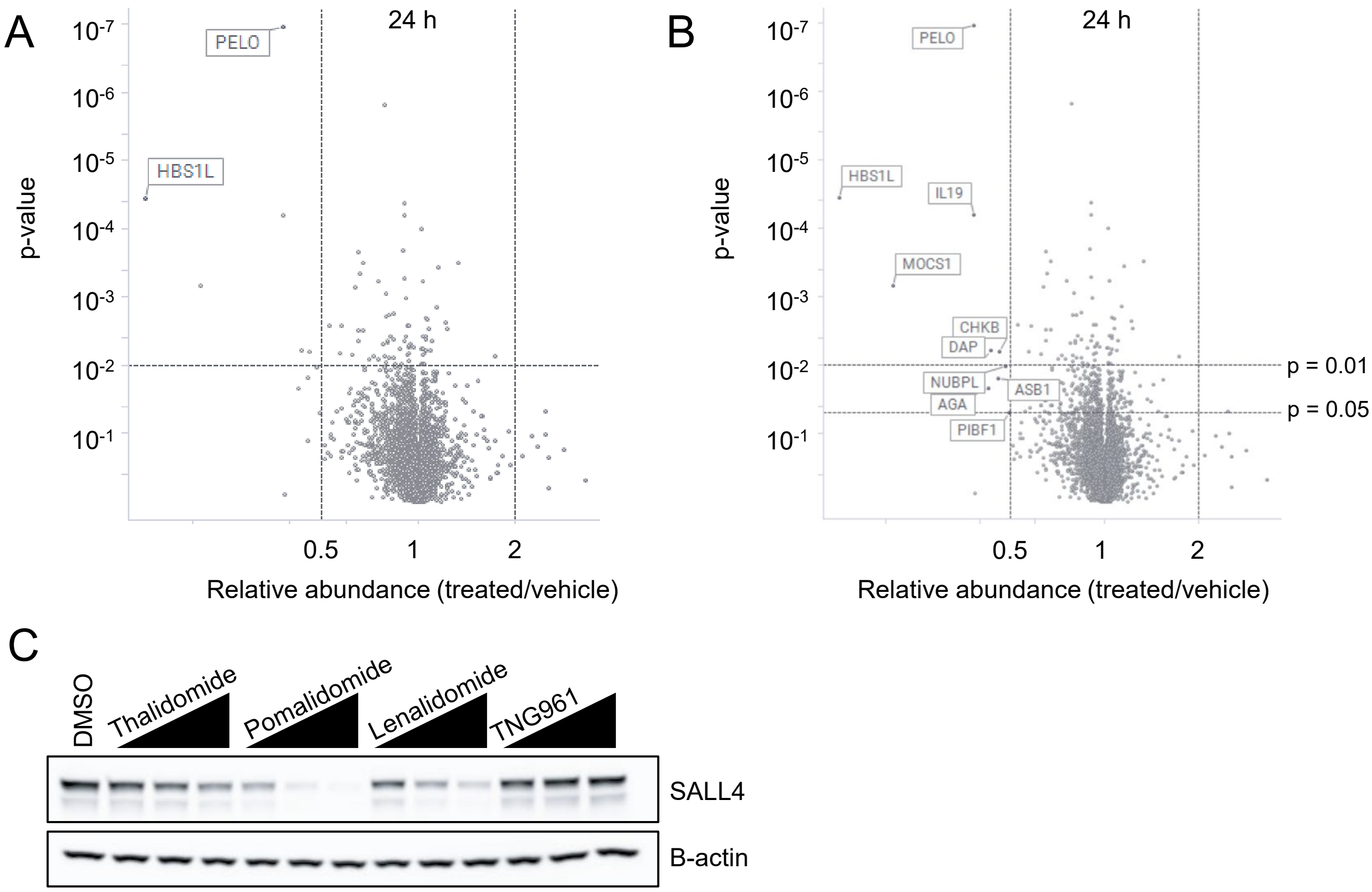

Supplementary Figure S2. TNG961 has proteome-wide selectivity for HBS1L. **A**, Global proteomics analysis of proteins in MM.1s cells after 24 h treatment with 100 nM TNG961 or DMSO. Data are presented as the relative abundance of protein in TNG961-treated vs. DMSO-treated cells. **B**, Data as in (**A**) with additional proteins labeled with p-value < 0.05 and relative abundance < 0.5. Beyond HBS1L and PELO, most of these proteins were only represented by one unique peptide or had less than 10% sequence coverage (IL19: 1 unique peptide identified, 10% protein coverage (relative abundance = 0.38, p = 0.00006); MOCS1: 1 unique peptide identified, 3% protein coverage (relative abundance = 0.21, p = 0.0007); DAP: 1 unique peptide identified, 25% protein coverage (relative abundance = 0.43, p = 0.006); CHKB: 2 unique peptides identified, 8% protein coverage (relative abundance = 0.46, p = 0.006)); NUBPL: 2 unique peptides identified, 12% protein coverage (relative abundance = 0.49, p = 0.01); ASB1: 1 unique peptide identified, 5% coverage (relative abundance = 0.46, p = 0.02); AGA: 1 unique peptide identified, 6% protein coverage (relative abundance = 0.43, p = 0.02); PIBF1: 2 unique peptides identified, 3% protein coverage (relative abundance = 0.5, p = 0.05)). Protein–protein interaction network analysis (STRING-db.org) does not indicate any known or predicted functional connections between these proteins and HBS1L/PELO. **C,** Western blot depicting unchanged SALL4 protein levels after 48 h treatment with 0.01, 0.1, or 1 μM TNG961 or IMiD-based degrader controls known to have SALL4 as a neosubstrate. Representative image of N=2 independent experiments.
