# Supplementary material for: TNG961 Is a Selective Oral HBS1L Molecular Glue Degrader for the Treatment of FOCAD-Deleted Cancers
Source: Cancer Discov. 2026 Apr 19;16(9):1884–902. doi: 10.1158/2159-8290.CD-26-0040 (PMC13531002; doi:10.1158/2159-8290.CD-26-0040)
Supplement: Supplementary file 3 [file cd-26-0040_figure_s3_suppsf3.pdf]

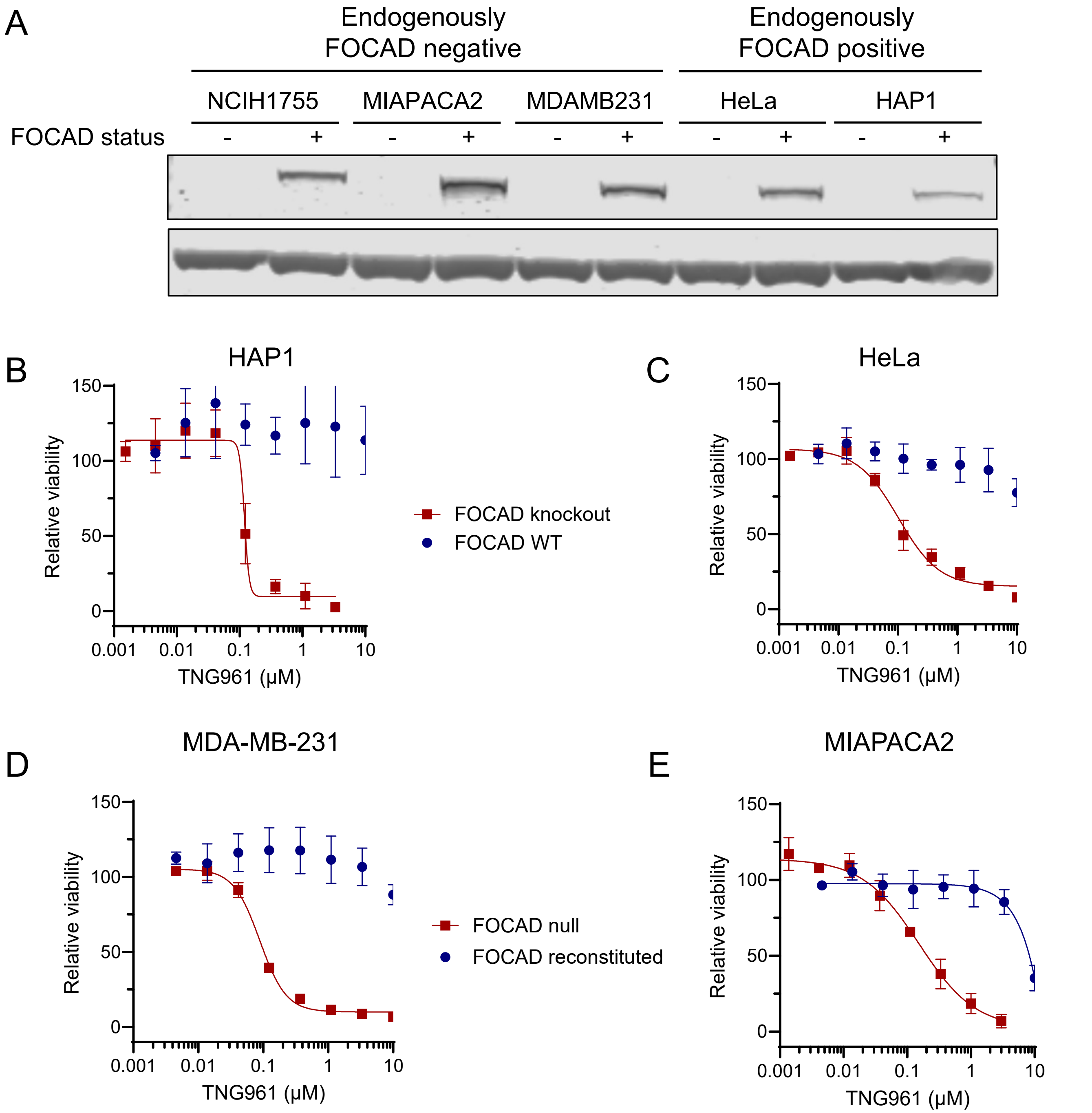

Supplementary Figure S3. TNG961 selectively inhibits growth of FOCAD negative cells. **A,** Immunoblots confirming generation of *FOCAD* isogenic pairs. HAP1 and HeLa are endogenously FOCAD-positive and isogenic pairs were generated by *FOCAD* knockout. MDA-MB-231 and MIAPACA2 are endogenously FOCAD-negative and isogenic pairs were generated by re-expression of full-length *FOCAD* cDNA. Representative image of N=2 biological replicates. **B-E**, Long-term viability assays in *FOCAD* isogenic pairs treated with the indicated doses of TNG961 and assessed by CellTiter-Glo after 7 days for HAP1 (**B**), HeLa (**C**), MDA-MB-231 (**D**) or after 14 days MIAPACA2 (**E**). Data represent mean ± SD for N=2-3 biological replicates.
