# Supplementary material for: TNG961 Is a Selective Oral HBS1L Molecular Glue Degrader for the Treatment of FOCAD-Deleted Cancers
Source: Cancer Discov. 2026 Apr 19;16(9):1884–902. doi: 10.1158/2159-8290.CD-26-0040 (PMC13531002; doi:10.1158/2159-8290.CD-26-0040)
Supplement: Supplementary file 4 [file cd-26-0040_figure_s4_suppsf4.pdf]

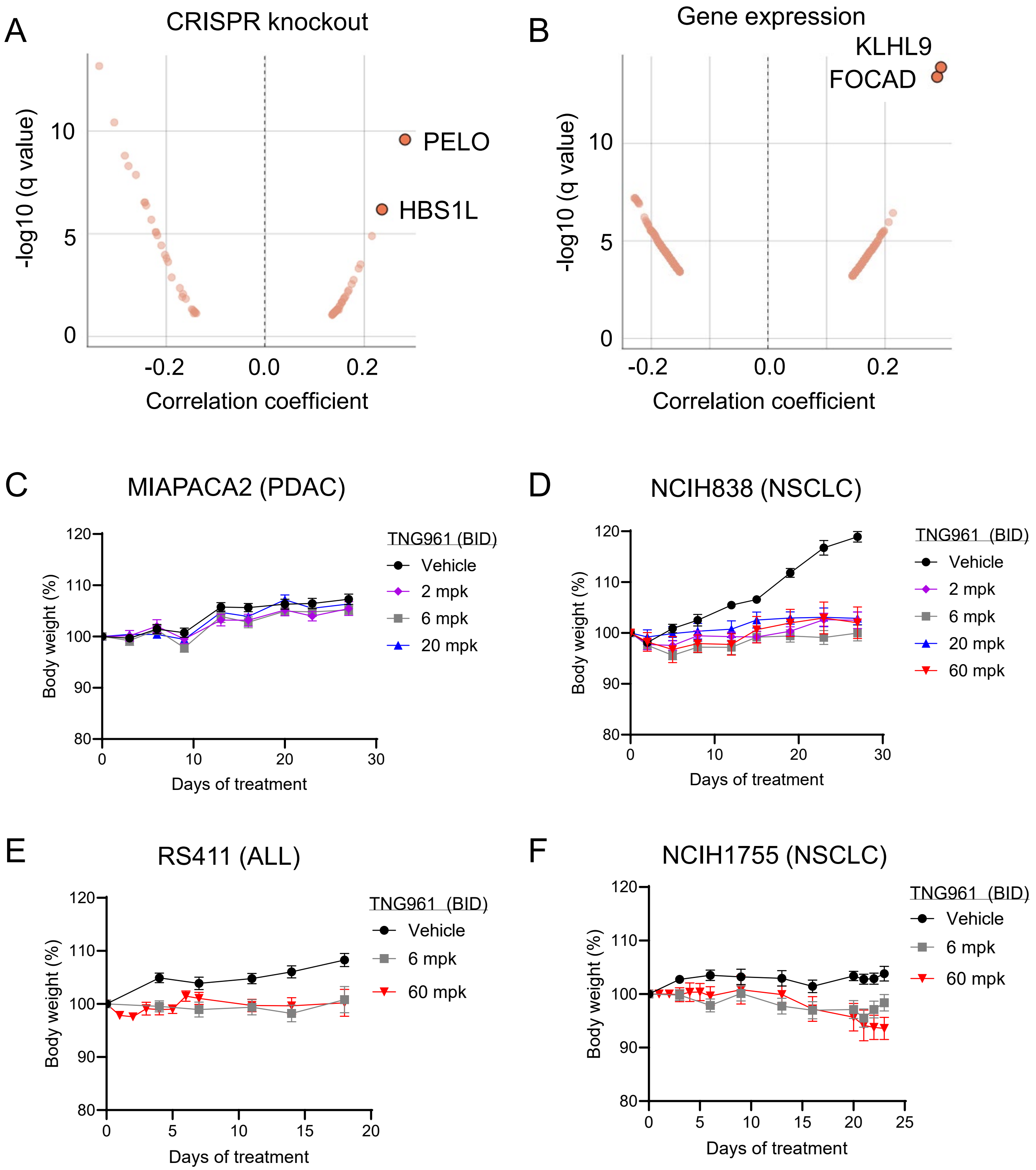

Supplementary Figure S4. TNG961 selectivity tracks with the HBS1L/PELO dependency signature and chromosome 9p21 loss and is well-tolerated. **A-B,** PRISM profiling across >900 cell lines showing top correlates of TNG961 sensitivity with (**A**) CRISPR knockout features and (**B**) gene expression features. **C-F**, Body weight monitoring for xenograft studies corresponding to **Figure 5E-H**, respectively. Data represent mean ± SEM for N=8 mice/arm.
