# Supplementary material for: TNG961 Is a Selective Oral HBS1L Molecular Glue Degrader for the Treatment of FOCAD-Deleted Cancers
Source: Cancer Discov. 2026 Apr 19;16(9):1884–902. doi: 10.1158/2159-8290.CD-26-0040 (PMC13531002; doi:10.1158/2159-8290.CD-26-0040)
Supplement: Supplementary file 5 [file cd-26-0040_figure_s5_suppsf5.pdf]

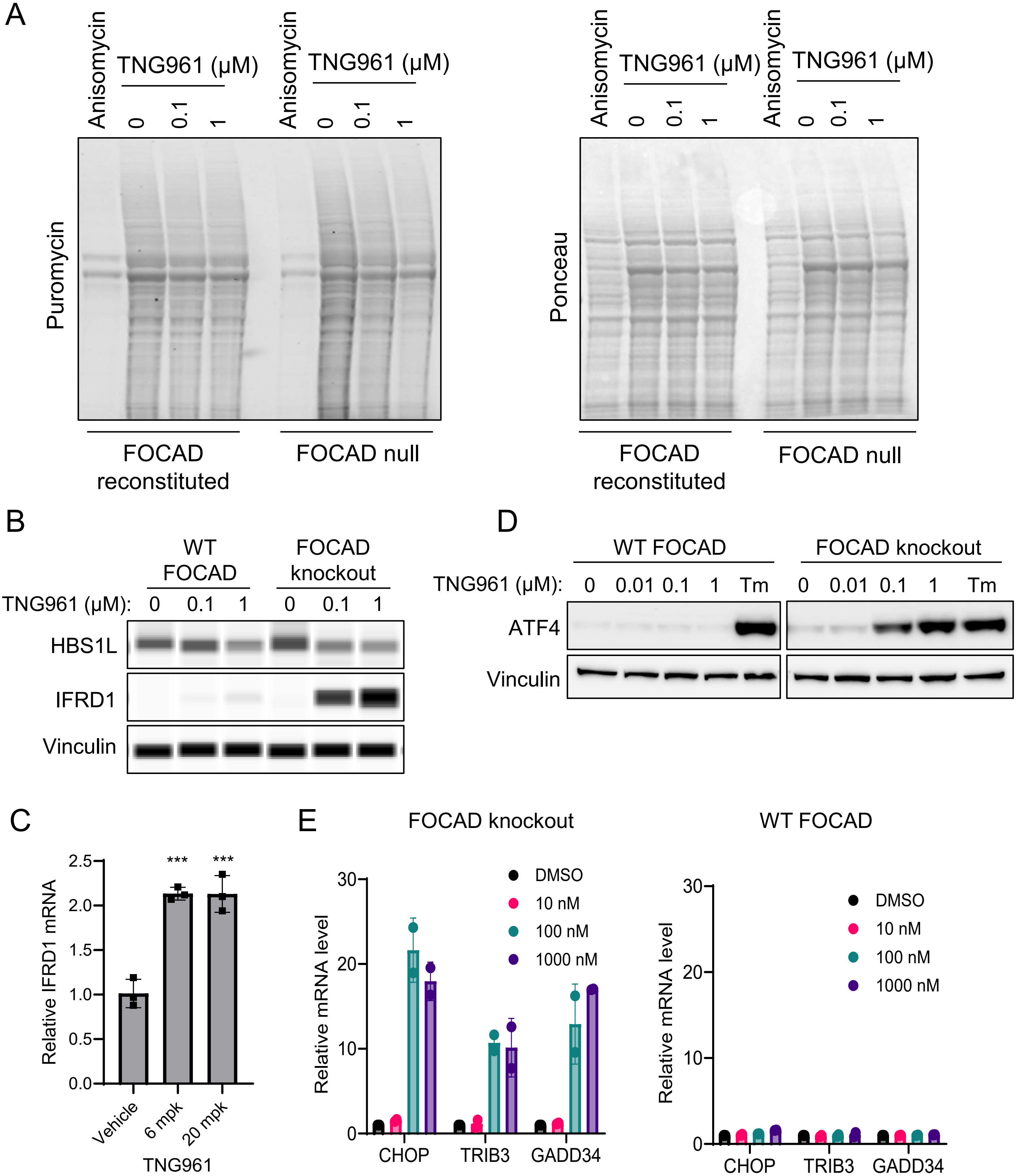

Supplementary Figure S5. TNG961 induces translational suppression and ER stress/UPR activation in a FOCAD-dependent manner in vitro and in vivo**.**

Legend continues on next page

Supplementary Figure S5. TNG961 induces translational suppression and ER stress/UPR activation in a FOCAD-dependent manner in vitro and in vivo**. A**, Representative puromycin incorporation immunoblot (translation readout; left side) and matched Ponceau staining (loading normalization; right side) from the translation assay in **Figure 6A**; representative image of N=3 independent experiments. **B,** HBS1L, IFRD1, and vinculin protein levels assessed by simple western in *FOCAD* isogenic HeLa cells after 48 h treatment with the indicated doses of TNG961. Representative images for N=2 independent experiments. **C**, Tumor qPCR analysis of IFRD1 levels after indicated TNG961 doses (BID) for 6 days. Tumors were isolated 12 hours post-last dose and preserved in RNAlater until analysis. Data represent mean ± SD for N=3 mice/arm. One-way ANOVA p=0.0002, ***p < 0.001, Dunnett's multiple comparisons test compared to vehicle. **D,** Immunoblot of ATF4 and vinculin in *FOCAD* isogenic HeLa cells after 48 h treatment with the indicated concentrations of TNG961. Tm, tunicamycin (positive control for ATF4 induction). Representative images for N=2 independent experiments. **E**, qPCR analysis of CHOP, TRIB3, and GADD34 in *FOCAD* isogenic HeLa cells after 48 h treatment with the indicated concentrations of TNG961. Bars show mean ± SD for N=2 independent experiments.
