# Supplementary material for: TNG961 Is a Selective Oral HBS1L Molecular Glue Degrader for the Treatment of FOCAD-Deleted Cancers
Source: Cancer Discov. 2026 Apr 19;16(9):1884–902. doi: 10.1158/2159-8290.CD-26-0040 (PMC13531002; doi:10.1158/2159-8290.CD-26-0040)
Supplement: Supplementary file 6 [file cd-26-0040_figure_s6_suppsf6.pdf]

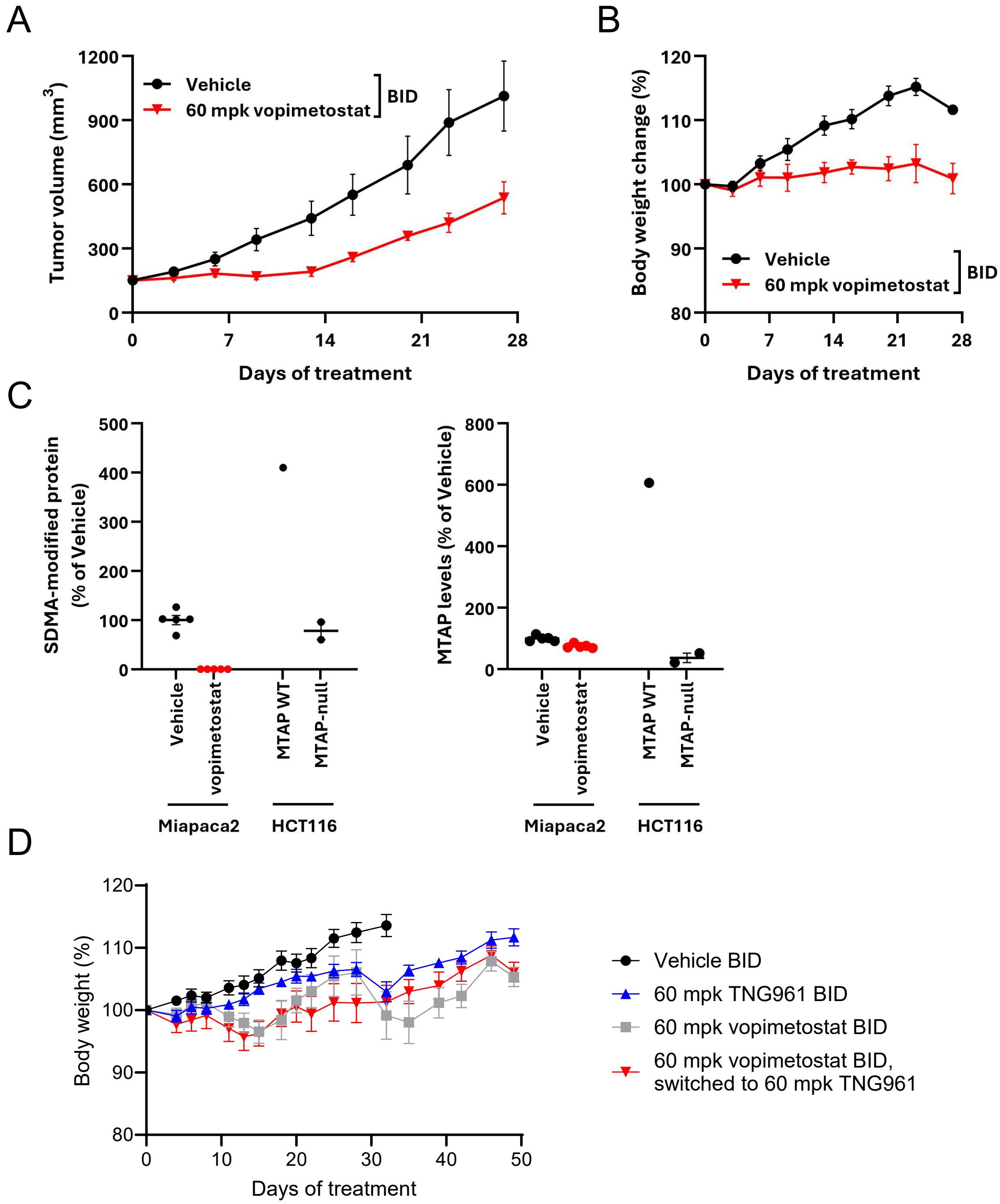

Supplementary Figure S6. TNG961 is active in PRMT5 inhibitor-refractory tumors harboring concomitant FOCAD/MTAP loss. **A,** Antitumor activity in MIAPACA2 xenografts treated with vopimetostat dosed as indicated (independent study). Data represent mean ± SEM for N=5 mice/arm **B,** Body weight monitoring corresponding to panel (A). Data represent mean ± SEM for N=5 mice/arm. **C,** Endpoint tumor immunoblots showing SDMA-modified proteins (left) and MTAP levels (right) from (A) confirming drug target engagement. HCT116 MTAP-isogenic tumors were used as controls for the SDMA and MTAP immunoblots (HCT116 MTAP WT and MTAP-null). **D,** Body weight monitoring for the study shown in main **Figure 7A** (including the vopimetostat to TNG961 switch arm, as indicated). Data are mean ± SEM from N=8 mice/arm.
