# Supplementary material for: TNG961 Is a Selective Oral HBS1L Molecular Glue Degrader for the Treatment of FOCAD-Deleted Cancers
Source: Cancer Discov. 2026 Apr 19;16(9):1884–902. doi: 10.1158/2159-8290.CD-26-0040 (PMC13531002; doi:10.1158/2159-8290.CD-26-0040)
Supplement: Supplementary file 7 [file cd-26-0040_table_s1_suppst1.docx]

**Supplementary Tables**

**TNG961 is a selective oral HBS1L molecular glue degrader for the treatment of FOCAD-deleted cancers**

Hilary E. Nicholson^1^*, Douglas A. Whittington^1^, Frank J. Bruzzese^1^, Katherine Lazarides^1^, Lauren Catherine M. Martires^1^, Matthew R. Tonini^1^, Helena N. Jenkins^1^, Minjie Zhang^1^, Preksha Shahagadkar^1^, Charlotte B. Pratt^1^, Kimberly J. Briggs^1^, Patrick McCarren^1^, Alice Tsai^1^, Madhavi Bandi^1^, Chengyin Min^1^, Alan Huang^1^, Hongxiang Zhang^1^, Samuel R. Meier^1^, Binzhang Shen^1^, Yi Yu^1^, Colin Liang^1^, Yong Liu^1^, Teng Teng^1^, John Zhang^1^, Adam Crystal^1^, William D. Mallender^1^, Xinyuan Edward Wu^1^, John P. Maxwell^1^, Jannik N. Andersen^1^

^1^Tango Therapeutics, 201 Brookline Avenue, Boston, MA 02215

***Corresponding author**: Hilary E. Nicholson, Tango Therapeutics, 201 Brookline Avenue, Suite 901, Boston, MA 02215,

Email: [hnicholson@tangotx.com](mailto:hnicholson@tangotx.com); Phone: 617-513-6837

**Running Title**: TNG961: an HBS1L degrader for treating FOCAD-deleted cancers

**Supplementary Table S1. Key reagents**

| **Reagent** | **Source** | **Catalog Number** | **Details for use** |
| --- | --- | --- | --- |
| MLN4924 | MedChemExpress | HY-70062 |  |
| CC-885 | MedChemExpress | HY-101488 |  |
| Lipofectamine | Thermo Fisher | L3000015 |  |
| Polybrene | Sigma Aldrich | TR-1003-G |  |
| MRT-2359 | Synthesized per Compound 145 in patent WO2022152821 | | |
| RPMI 1640 | Gibco | 11875093 |  |
| DMEM | Gibco | 11965-084 |  |
| EMEM | Gibco | 11095072 |  |
| Fetal Bovine Serum | Gemini Bio Products | 100-500 |  |
| Sodium pyruvate | Thermo Fisher | 11360070 |  |
| L-glutamine | Thermo Fisher | 25030081 |  |
| Blasticidin | Thermo Fisher | A1113903 | 10 µg/mL |
| Puromycin | Thermo Fisher | A1113803 | 2 µg/mL |
| Neomycin | Thermo Fisher | 10131027 | 200-400 µg/mL |
| HBS1L antibody | Proteintech | 10359-1-AP (RRID AB_2114730) | 1:1000 |
| PELO antibody | Abcam | ab154335 | 1:1000 |
| CRBN antibody | Abcam | ab244223 (RRID AB_3714645) | 1:1000 |
| SALL4 antibody | Cell Signaling | 5850 (RRID AB_10858883) | 1:1000 |
| GSPT1 antibody | Cell Signaling | 14980 (RRID AB_2798677) | 1:1000 |
| FOCAD antibody | Proteintech | 30140-1-AP (RRID AB_3086244) | 1:600 |
| IFRD1 antibody | Abcam | ab137633 | 1:1000 |
| ATF4 antibody | Cell Signaling | 11815S | 1:500 |
| HA antibody | Cell Signaling | 2367S | 1:1000 |
| SKIV2L antibody | Proteintech | 11462-1-AP (RRID AB_2187472) | 1:1000 |
| Puromycin antibody | Sigma Aldrich | MABE343 (RRID AB_2566826) | 1:1000 |
| XBP1s antibody | Cell Signaling | 12782 (RRID AB_2687943) | 1:1000 |
| XBP1u antibody | Abcam | ab37152 (RRID AB_778939) | 1:500 |
| Actin antibody | Cell Signaling | 3700 (RRID AB_2242334) | 1:5000 |
| Vinculin antibody | Cell Signaling | 13901 (RRID AB_2728768) | 1:10,000 |
| GAPDH antibody | Proteintech | 10494-1-AP (RRID AB_2263076) | 1:5000 |
| Goat anti-rabbit IR680 | LI-COR | 926-68071 | 1:15,000 |
| Goat anti-mouse | LI-COR | 926-32210 | 1:15,000 |
| CHOP qPCR probe | Life Technologies | Hs00358796_g1 |  |
| TRIB3 qPCR probe | Life Technologies | Hs01082394_m1 |  |
| GADD34 qPCR probe | Life Technologies | Hs000169585_m1 |  |
| FOCAD qPCR probe (exon 1-2) | Life Technologies | Hs.PT. 58.39883457.gs |  |
| FOCAD qPCR probe (exon 15-16) | Life Technologies | Hs.PT.58. 19255669 |  |
| FOCAD qPCR probe (exon 22-23) | Life Technologies | Hs.PT.58. 28343308 |  |
| FOCAD qPCR probe (exon 23-24) | Life Technologies | Hs.PT.58. 28343308 |  |
| FOCAD qPCR probe (exon 26-27) | Life Technologies | Hs.PT.58.39578246 |  |
| FOCAD qPCR probe (exon 44) | Life Technologies | Hs.PT.58.40719232.g |  |
| Human RPLPO-VIC/MGB | Life Technologies | 4326314E |  |
| Streptavidin-HRP | Protein Simple | 042-976 | For simple western |
| Luminol-S | Protein Simple | 043-311 | For simple western |
| Peroxide | Protein Simple | 043-379 | For simple western |
| Anti-mouse secondary antibody | Protein Simple | 042-205 | For simple western |
| Anti-rabbit secondary antibody | Protein Simple | 042-206 | For simple western |
| RIPA lysis buffer | Thermo Fisher | 89900 |  |
| Halt protease inhibitor | Thermo Fisher | 1861281 |  |
| Pierce™ Rapid Gold BCA Protein Assay Kit | Thermo Fisher | A53225 |  |
| Lonza 4D-Nucleofector™ X Unit (Program CM-130) | Lonza | AAF-1003X |  |
| SF Cell Line 4D-Nucleofector™ and X Kit S | Lonza | V4XC-2032 |  |
| TrueCut.v2 Cas9 protein | Thermo Fisher | A36499 |  |
| IDT Alt-R® Cas9 Electroporation Enhancer | Integrated DNA Technologies | 1075915 |  |
| NanoGlo HiBiT lytic detection reagent | Promega | N3040 |  |
