# Supplementary material for: TNG961 Is a Selective Oral HBS1L Molecular Glue Degrader for the Treatment of FOCAD-Deleted Cancers
Source: Cancer Discov. 2026 Apr 19;16(9):1884–902. doi: 10.1158/2159-8290.CD-26-0040 (PMC13531002; doi:10.1158/2159-8290.CD-26-0040)
Supplement: Supplementary file 8 [file cd-26-0040_table_s2_suppst2.docx]

**Supplementary Table S2. Cell lines and culture conditions**

| **Cell line** | **Source** | **Base media** | **Media supplements** |
| --- | --- | --- | --- |
| MM.1s | ATCC (CRL-2974) (RRID CVCL_8792) | RPMI 1640 | 10% FBS |
| MIAPACA2 | ATCC (CRL-1420) (RRID CVCL_0428) | DMEM | 10% FBS |
| HEK293 LgBiT Cell Line | Promega (N2672) | DMEM | 10% FBS + 1 mM sodium pyruvate + 2 mM L-glutamine |
| GSPT1 HiBiT knock-in HEK293 (LgBiT) | Promega (CS3023106) | DMEM | 10% FBS + 1 mM sodium pyruvate |
| NCIH1755 | ATCC (CRL-5892) (RRID CVCL_1492) | RPMI 1640 | 10% FBS |
| HAP1 | Horizon (C631) | EMEM | 10% FBS |
| HAP1 FOCAD knockout | Horizon (HZGHC008250c008) | EMEM | 10% FBS |
| HeLa | Abcam (ab255928) (RRID CVCL_0030) | EMEM | 10% FBS |
| HeLa FOCAD knockout | Abcam (ab265627) (RRID CVCL_B1S9) | EMEM | 10% FBS |
| MDA-MB-231 | ATCC (CRM-HTB-26) (RRID CVCL_0062) | DMEM | 10% FBS |
| NCIH838 | ATCC (CRL-5844) (RRID CVCL_1594) | RPMI 1640 | 10% FBS |
| RS411 | ATCC (CRL-1873) (RRID CVCL_0093) | RPMI 1640 | 10% FBS |
| Kelly | WuXi AppTec (RRID CVCL_2092) | RPMI 1640 | 10% FBS |
