# Supplementary material for: TNG961 Is a Selective Oral HBS1L Molecular Glue Degrader for the Treatment of FOCAD-Deleted Cancers
Source: Cancer Discov. 2026 Apr 19;16(9):1884–902. doi: 10.1158/2159-8290.CD-26-0040 (PMC13531002; doi:10.1158/2159-8290.CD-26-0040)
Supplement: Supplementary file 9 [file cd-26-0040_table_s3_suppst3.docx]

**Supplementary Table S3. cDNA, sgRNA, and other DNA/RNA sequences**

| **Reagent** | **Encoded DNA/RNA** | **DNA/RNA sequence** |
| --- | --- | --- |
| crRNA | HiBiT knock-in crRNA | CCGATGCCGGGCCATGACGG |
| Donor template | HiBiT knock-in donor template (C-terminal) | TTCCCTTTGTTGTTCTTATAATGTGATTGATTAAATTACAGCAAATGTCTTTTTTTTTTTTGTTTTAACAGATAAAAGAAGTCTCCGTGAGCGGCTGGCGGCTGTTCAAGAAGATTAGCTGATGGGTCAGAATTTCTAACACGTTTCTGGATACAGTGAAATAGCTAACCTCTGTTTCAAGAATGCAGTTATTAAGTCA |
| Donor template | HiBiT knock-in donor template (N-terminal) | GTTGGCGCCTTATCAGTATCTAAGCGGAGTGTTTTGGAAGGAGTTAAGGGGCTGTGGCAAACGCCCTCTCCGCCGTCATGGTCTCCGTGAGCGGCTGGCGGCTGTTCAAGAAGATTAGCGCCCGGCATCGGAATGTTCGAGGCTATAACTACGATGAAGGTAGGTGGCAGGGTCATGCCGGGACGTTCTTTTCCCGGCT |
| pTG3267 | sgCRBN | GCACGATGACGACAGCTGTC |
| pTG59 | sgITC (intron-cutting control) | GTACATGAAAAGGCTCTAGG |
| pTG2904 | sgHBS1L | TTGTTTAAGGGGAGTAACCA |
| pTG5729 | sgGSPT1 | ATCAGTTGATGGACCAATC |
| pTG185 | Cas9 cDNA | GACAAGAAGTACAGCATCGGCCTGGACATCGGCACCAACTCTGTGGGCTGGGCCGTGATCACCGACGAGTACAAGGTGCCCAGCAAGAAATTCAAGGTGCTGGGCAACACCGACCGGCACAGCATCAAGAAGAACCTGATCGGAGCCCTGCTGTTCGACAGCGGCGAAACAGCCGAGGCCACCCGGCTGAAGAGAACCGCCAGAAGAAGATACACCAGACGGAAGAACCGGATCTGCTATCTGCAAGAGATCTTCAGCAACGAGATGGCCAAGGTGGACGACAGCTTCTTCCACAGACTGGAAGAGTCCTTCCTGGTGGAAGAGGATAAGAAGCACGAGCGGCACCCCATCTTCGGCAACATCGTGGACGAGGTGGCCTACCACGAGAAGTACCCCACCATCTACCACCTGAGAAAGAAACTGGTGGACAGCACCGACAAGGCCGACCTGCGGCTGATCTATCTGGCCCTGGCCCACATGATCAAGTTCCGGGGCCACTTCCTGATCGAGGGCGACCTGAACCCCGACAACAGCGACGTGGACAAGCTGTTCATCCAGCTGGTGCAGACCTACAACCAGCTGTTCGAGGAAAACCCCATCAACGCCAGCGGCGTGGACGCCAAGGCCATCCTGTCTGCCAGACTGAGCAAGAGCAGACGGCTGGAAAATCTGATCGCCCAGCTGCCCGGCGAGAAGAAGAATGGCCTGTTCGGAAACCTGATTGCCCTGAGCCTGGGCCTGACCCCCAACTTCAAGAGCAACTTCGACCTGGCCGAGGATGCCAAACTGCAGCTGAGCAAGGACACCTACGACGACGACCTGGACAACCTGCTGGCCCAGATCGGCGACCAGTACGCCGACCTGTTTCTGGCCGCCAAGAACCTGTCCGACGCCATCCTGCTGAGCGACATCCTGAGAGTGAACACCGAGATCACCAAGGCCCCCCTGAGCGCCTCTATGATCAAGAGATACGACGAGCACCACCAGGACCTGACCCTGCTGAAAGCTCTCGTGCGGCAGCAGCTGCCTGAGAAGTACAAAGAGATTTTCTTCGACCAGAGCAAGAACGGCTACGCCGGCTACATTGACGGCGGAGCCAGCCAGGAAGAGTTCTACAAGTTCATCAAGCCCATCCTGGAAAAGATGGACGGCACCGAGGAACTGCTCGTGAAGCTGAACAGAGAGGACCTGCTGCGGAAGCAGCGGACCTTCGACAACGGCAGCATCCCCCACCAGATCCACCTGGGAGAGCTGCACGCCATTCTGCGGCGGCAGGAAGATTTTTACCCATTCCTGAAGGACAACCGGGAAAAGATCGAGAAGATCCTGACCTTCCGCATCCCCTACTACGTGGGCCCTCTGGCCAGGGGAAACAGCAGATTCGCCTGGATGACCAGAAAGAGCGAGGAAACCATCACCCCCTGGAACTTCGAGGAAGTGGTGGACAAGGGCGCTTCCGCCCAGAGCTTCATCGAGCGGATGACCAACTTCGATAAGAACCTGCCCAACGAGAAGGTGCTGCCCAAGCACAGCCTGCTGTACGAGTACTTCACCGTGTATAACGAGCTGACCAAAGTGAAATACGTGACCGAGGGAATGAGAAAGCCCGCCTTCCTGAGCGGCGAGCAGAAAAAGGCCATCGTGGACCTGCTGTTCAAGACCAACCGGAAAGTGACCGTGAAGCAGCTGAAAGAGGACTACTTCAAGAAAATCGAGTGCTTCGACTCCGTGGAAATCTCCGGCGTGGAAGATCGGTTCAACGCCTCCCTGGGCACATACCACGATCTGCTGAAAATTATCAAGGACAAGGACTTCCTGGACAATGAGGAAAACGAGGACATTCTGGAAGATATCGTGCTGACCCTGACACTGTTTGAGGACAGAGAGATGATCGAGGAACGGCTGAAAACCTATGCCCACCTGTTCGACGACAAAGTGATGAAGCAGCTGAAGCGGCGGAGATACACCGGCTGGGGCAGGCTGAGCCGGAAGCTGATCAACGGCATCCGGGACAAGCAGTCCGGCAAGACAATCCTGGATTTCCTGAAGTCCGACGGCTTCGCCAACAGAAACTTCATGCAGCTGATCCACGACGACAGCCTGACCTTTAAAGAGGACATCCAGAAAGCCCAGGTGTCCGGCCAGGGCGATAGCCTGCACGAGCACATTGCCAATCTGGCCGGCAGCCCCGCCATTAAGAAGGGCATCCTGCAGACAGTGAAGGTGGTGGACGAGCTCGTGAAAGTGATGGGCCGGCACAAGCCCGAGAACATCGTGATCGAAATGGCCAGAGAGAACCAGACCACCCAGAAGGGACAGAAGAACAGCCGCGAGAGAATGAAGCGGATCGAAGAGGGCATCAAAGAGCTGGGCAGCCAGATCCTGAAAGAACACCCCGTGGAAAACACCCAGCTGCAGAACGAGAAGCTGTACCTGTACTACCTGCAGAATGGGCGGGATATGTACGTGGACCAGGAACTGGACATCAACCGGCTGTCCGACTACGATGTGGACCATATCGTGCCTCAGAGCTTTCTGAAGGACGACTCCATCGACAACAAGGTGCTGACCAGAAGCGACAAGAACCGGGGCAAGAGCGACAACGTGCCCTCCGAAGAGGTCGTGAAGAAGATGAAGAACTACTGGCGGCAGCTGCTGAACGCCAAGCTGATTACCCAGAGAAAGTTCGACAATCTGACCAAGGCCGAGAGAGGCGGCCTGAGCGAACTGGATAAGGCCGGCTTCATCAAGAGACAGCTGGTGGAAACCCGGCAGATCACAAAGCACGTGGCACAGATCCTGGACTCCCGGATGAACACTAAGTACGACGAGAATGACAAGCTGATCCGGGAAGTGAAAGTGATCACCCTGAAGTCCAAGCTGGTGTCCGATTTCCGGAAGGATTTCCAGTTTTACAAAGTGCGCGAGATCAACAACTACCACCACGCCCACGACGCCTACCTGAACGCCGTCGTGGGAACCGCCCTGATCAAAAAGTACCCTAAGCTGGAAAGCGAGTTCGTGTACGGCGACTACAAGGTGTACGACGTGCGGAAGATGATCGCCAAGAGCGAGCAGGAAATCGGCAAGGCTACCGCCAAGTACTTCTTCTACAGCAACATCATGAACTTTTTCAAGACCGAGATTACCCTGGCCAACGGCGAGATCCGGAAGCGGCCTCTGATCGAGACAAACGGCGAAACCGGGGAGATCGTGTGGGATAAGGGCCGGGATTTTGCCACCGTGCGGAAAGTGCTGAGCATGCCCCAAGTGAATATCGTGAAAAAGACCGAGGTGCAGACAGGCGGCTTCAGCAAAGAGTCTATCCTGCCCAAGAGGAACAGCGATAAGCTGATCGCCAGAAAGAAGGACTGGGACCCTAAGAAGTACGGCGGCTTCGACAGCCCCACCGTGGCCTATTCTGTGCTGGTGGTGGCCAAAGTGGAAAAGGGCAAGTCCAAGAAACTGAAGAGTGTGAAAGAGCTGCTGGGGATCACCATCATGGAAAGAAGCAGCTTCGAGAAGAATCCCATCGACTTTCTGGAAGCCAAGGGCTACAAAGAAGTGAAAAAGGACCTGATCATCAAGCTGCCTAAGTACTCCCTGTTCGAGCTGGAAAACGGCCGGAAGAGAATGCTGGCCTCTGCCGGCGAACTGCAGAAGGGAAACGAACTGGCCCTGCCCTCCAAATATGTGAACTTCCTGTACCTGGCCAGCCACTATGAGAAGCTGAAGGGCTCCCCCGAGGATAATGAGCAGAAACAGCTGTTTGTGGAACAGCACAAGCACTACCTGGACGAGATCATCGAGCAGATCAGCGAGTTCTCCAAGAGAGTGATCCTGGCCGACGCTAATCTGGACAAAGTGCTGTCCGCCTACAACAAGCACCGGGATAAGCCCATCAGAGAGCAGGCCGAGAATATCATCCACCTGTTTACCCTGACCAATCTGGGAGCCCCTGCCGCCTTCAAGTACTTTGACACCACCATCGACCGGAAGAGGTACACCAGCACCAAAGAGGTGCTGGACGCCACCCTGATCCACCAGAGCATCACCGGCCTGTACGAGACACGGATCGACCTGTCTCAGCTGGGAGGCGAC |
| pTG3033/pTG3044 | FOCAD cDNA | ATGTCAGATGATATCAGGAAAAGGTTTGAATTTCCAAATTCTCTTATCCAATCACAGGCTGTGGGTCATCTTATTGCTGCAGTCCTAAAGGAAAATGGTTTTTCAGAAAAGATTCACCAATCTACAAATCAGACTCCTGCTTTGAACTTGCTGTGGGAGAAGTGTTGCAGTGACAATGTAGTGGTTCGAACAGCCTGCTGTGAAGGTCTGGTGGCACTCGTTGCTCAGGATCATGCAGAGTTCAGCTATGTTCTCAATGGGATACTCAACTTGATTCCATCAACCAGAAATACACATGGCTTGATAAAAGCCATTATGCACTTACTACAAATGCAAGCTCTTAAGGAAGGACAAGGTGGGGAAAAGAATATTCAGAGTATATATACCATTAGAAATCATCCTCATCCTTTGATAACTGTGCTTGAACACAGACCTGATTGCTGGCCAGTGTTTTTGCAGCAGCTGACAGCGTTTTTCCAGCAGTGCCCTGAAAGGTTAGAAGTTTCATGCATTCAAATAATGGCACCATTTCTGTGGTATCTGTATTGTGAACCATCTCAGTTACAAGAATATGCTAAACTCCGACTAGCCCTGCTGAAAGTCTTACTTCAACCCCAGGTTCTTTGTGACAAAGATCAACCATCAATACTGGAACAGCAGATACTTCAACTGTGTTGTGACATAGTTCCATGTTTGCAGGTAAAAGATTTGATACAGACAACAGAGGCGATGATGTTTATTGAGGAAGTATGTTTAAGCCTTTTGCGTCATCCTGTTTTCTGGAAAATTCAGCTTACCCAGATGAGTCTTCAGCTGCTGTGTGTCAGTGAAGTCAGCTTAAAGATAACTGGTGAATGTTCATCTTCAATTCACCTTTTAGAGCACAGTGTTGAACTTCTGAAGGAGGATTTTCCTGTTGAACTGGTCATAATTGGAATAGCTTTACTACTTCTACAGACTCCAGCAAGTCAGCAGAAGCCAATCTTAAATCTAGCTTTGAAGCTCCTCTCTGTTACTGAGGATCAGAAAATCCCAAAGTCCTCTCTGCTGCTAGTGATGCCAATTCTGCAGATACTATCTTCTACTGCCTTGGAAGACTGTATATCTGTGGATGAAGAAGGTCCCTCTAGGCAGCAGTTGGCTCTAAACCTTTTGGAAATGATACAGCAGGAATGTTACAGAGATGACCACCAAAAGCTCTCCTACAAGCTTGTGTGCCCTGTAACCAGTATGTATGGTACAATATTTACAGCCTGGAGGATTCTTGAAGTAATGACAGACTCGTCTGCTGCAAGTGACTGGTTGGCTTCAGTAGAGTCATTGCTTCCTATTACTGCTGTGATCCCTGCGCCTGCCTTTCTTCTGCTGGCTCACCTCCTTGTTGAAGACAAAGGACAAAATCTTCACCAAATACTCAAGGTCACTACAGAATTAGCCCAAGCAGATTCCTCCCAGGTGCCAAATCTGATTCCAGTTTTGATGTTCAAATTGGGAAGACCACTGGAACCTATATTATATAATGATATATTGTATACTTTACCTAAGCTTGGTGTTCACAAGGTGTGTATAGGACAAATTCTACGAATAATACAACTACTTGGAACCACACCACGACTAAGAGCTGTCACTTTGCGCTTGCTGACATCTTTGTGGGAAAAGCAGGACCGAGTCTATCCTGAACTGCAGCGTTTCATGGCTGTGTCTGATGTACCTTCTCTTTCGGTGGGCAAGGAAGTCCAATGGGAGAAACTGATTGCAAAAGCAGCATCAATCAGAGATATATGTAAGCAGAGGCCATATCAACATGGTGCAGATATGTTGGCAGCTATTTCTCAAGTGTTGAATGAATGCACCAAGCCTGATCAAGCTACTCCAGCAGCCTTGGTATTACAGGGTCTTCATGCACTCTGTCAAGCTGAGGTTGTTTGCATTCGCTCCACTTGGAATGCTCTCTCTCCAAAGCTGAGTTGTGACACAAGACCTCTCATTCTGAAGACACTGAGTGAACTATTTTCTCTAGTTCCTTCCTTAACGGTCAATACAACTGAATATGAGAATTTTAAAGTTCAAGTCCTCAGCTTCCTCTGGACTCATACTCAAAACAAGGACCCAATTGTAGCAAATGCTGCATATAGATCCCTGGCCAACTTTAGTGCAGGAGAACACACCATTCTTCATCTGCCTGAAAAGATAAGACCAGAAATTCCCATTCCTGAAGAGTTAGATGACGATGAAGATGTTGAGGATGTGGATCTTTCAGTTCCTGGCTCTTGCTATCTCAAACTGTTGTCACTCACTCCCCCTTTGGTTTTACCAGCTTTGGAGGAATTTTTTACATCACTTGTGAAGCAAGAAATGGTGAATATGCCTCGTGGGATATATCACTCTGCATTAAAAGGAGGTGCCCGCTCAGACCAAGGAAAGACTGTAGCAGGAATCCCCAATTTTATATTGAAAATGTATGAAACAAACAAGCAACCAGGACTGAAACCTGGCCTTGCAGGTGGTATGTTATTTTGCTATGATGTTTCCATGTATCAGAGTAAAGATGGAAAACCATTGAACAGACTGATGGCCAGCAGAGGGCGAAGTTTCAAGCAGACTTCACTTGCTCTTGTACATGAGGTTCATATCCAGCTTTCAGAGTGGCACCGTGCAATTTTTCTTCCACAGGCCTGGCTTGCATACATGAATCGAGCTTATCATGCCATTTTACAGGGAAGACTAGGAGAGCTGGAGTTGCAGTTAAAACATGGAAAAGAAGAACCTGAGGAGGTGCAGTACAAAAAAAGCACAGCCTGGCTCTGGGTTAGAGACATGCTGACTGATGAGATCACCAAGGCAGCTGCAAAGGAGAGTCCGGTAGTGAAAGGCAATGCGCTGTTAGCTCTAAGCAGCCTTGCTGTCGTCGTATCTAGACATGAAGCCAGCCTCTCCTCAGACTCTGACGGGCTCCTGGAGGTTCAACCTAATTTCCTTTCAATGAAAGAGTGGGTTTCCATGGTACTTGATACACTCTTGGTCATTGTGGATAGCCATTACCAACCCAGAGGGCAACTTCTCTCCTGGTTTTATTATAAGTCCTATTCTGGTGAAAACACAGCTAGTGCCATTGCCCGTTCTGCTGCCGCCACGGCTTTGTCTCTCCTTGTGCCAGTTTTCATTATCTCTTGCAAAGAGAAGGTTGAGGAAATCCTGAACATGCTGACTGCCAGGTTACCTGGGAAACCAAGTGCTGATGAGTCTCAAGCCGTGCAAATCCACATGGGCCTTGCTTTAGGGATGTTTCTCTCTCGCTTGTGTGAAGAGAAACTCAGTGATATATCTGGCCAAGAGATGAACCTTCTTCTGATGAAGTCGTTGGATGCCCTGGAAAATTGCTGCTTTGACACTAGTCTTGAATACAACACGGGCTGTATATTGGGAGTTGGACTTGTTCTGTCCCTCATGAGCCACAGCAGCCAAATGCAGTCCCGCGTTCACGTAGCAGCATTGCTCCGGAAGCTGTCTGCGCACGTAGATGACAGCGGGAGCCAGAGCAGAACGTTTCAGGAGGTCCTTGCCTACACACTTAGCTGTGTATGTACATCAGCGTTCAGTGCTGGAATTATTGAGGCTACAGAGGCTGAGGATGTTATGAACAAGCTTCGACTGTTAGTGGAGAATAGCCAGCAGACTTCAGGTTTTGCCCTGGCTTTAGGAAACATAGTTCATGGATTGTCTGTGTGTGGACATGGAAAAGCTGAAGACTTGGGCAGCAAACTACTCCCTGCCTGGATCAGAATTGTTCTAACAGAGGGCACTCCCACAATGCTTTGTCTGGCAGCTCTTCATGGCATGGTGGCCTTGGTAGGCTCTGAAGGGGATGTAATGCAGCTGAAATCAGAAGCCATCCAGACCTCTCATTTTCAAGGCAGACTTAATGAAGTCATTAGAACCTTAACTCAGGTCATTAGTGTCTCTGGGGTGATTGGTCTCCAGTCAAATGCAGTCTGGCTTCTTGGACATCTTCATCTATCTACTCTATCCTCAAGTCAAAGTAGAGCCTCTGTTCCTACTGACTATAGCTACTTGCCTGAAAGCAGTTTTATTGGAGCAGCTATTGGCTTCTTCATTACAGGAGGAAAAAAAGGTCCTGAATCTGTGCCTCCTTCCCTTCTTAAAGTAGTGATGAAACCCATAGCAACTGTTGGAGAAAGCTACCAATATCCTCCTGTGAACTGGGCTGCACTTCTCTCTCCACTTATGAGGCTAAATTTTGGTGAAGAGATCCAGCAACTGTGCCTTGAAATTATGGTGACCCAGGCACAGTCATCCCAGAATGCAGCTGCACTATTGGGCTTGTGGGTGACACCACCACTGATCCACAGTCTGAGTCTGAATACCAAGAGATATCTCCTGATATCTGCACCTCTGTGGATAAAACACATCTCTGATGAACAGATCCTGGGTTTTGTTGAAAATTTAATGGTGGCAGTTTTTAAAGCAGCTTCCCCACTTGGAAGTCCTGAGCTATGCCCAAGTGCTTTACACGGTCTGAGCCAGGCCATGAAACTGCCCAGCCCTGCCCACCACCTCTGGAGTCTGCTCTCTGAAGCTACTGGGAAAATTTTTGACCTCCTGCCAAATAAGATTCGGAGAAAGGATCTAGAGCTGTATATCAGCATAGCAAAATGCCTCTTAGAAATGACAGATGATGATGCCAATCGGATCGCCCAGGTTACTAAGAGCAACATAGAAAAAGCTGCCTTTGTCAAACTGTACTTAGTCTCTCAAGGACGATTCCCCTTGGTGAACCTGACCGATATGCTGAGCGTTGCTGTGCAGCACCGTGAGAAAGAGGTGTTGGCCTGGATGATTCTGCACAGCTTATACCAGGCACGGATTGTGAGCCATGCCAATACGGGCGTTTTGAAGAGAATGGAGTGGCTCTTGGAACTGATGGGTTATATTAGAAATGTTGCTTACCAGTCAACATCCTTTCACAATACGGCTCTTGACAAGGCTTTGGACTTCTTCTTGCTGATATTTGCAACCGCAGTGGTTGCATGGGCTGACCACACTGCCCCTCTCCTCCTCGGCCTCAGTGCCAGTTGGTTGCCATGGCATCAGGAGAATGGCCCGGCTGGGCCAGTACCAAGCTTCCTTGGCAGGAGTCCAATGCACAGGGTCACTCTGCAGGAGGTTCTCACTCTCCTTCCCAATAGCATGGCTCTGCTGCTGCAGAAAGAGCCATGGAAGGAACAGACCCAGAAGTTCATTGACTGGCTATTCAGCATCATGGAAAGCCCTAAAGAAGCCCTCTCAGCACAGTCCAGGGATCTTTTGAAAGCCACCCTGCTGTCCTTGAGAGTTCTCCCAGAGTTTAAGAAGAAAGCTGTATGGACCAGAGCATATGGTTGGTGA |
| pTG3080 | HBS1L G625N | GCCCGGCATCGGAATGTTCGAGGCTATAACTACGATGAAGATTTTGAAGATGATGATCTCTACGGCCAGTCTGTAGAGGATGATTATTGTATTTCGCCGTCAACAGCTGCTCAGTTTATTTATTCACGGCGTGACAAACCTTCCGTTGAGCCTGTGGAAGAATATGATTATGAAGATCTGAAAGAATCTTCCAATTCTGTTTCAAACCATCAGCTCAGTGGATTTGATCAAGCTCGTCTTTATTCCTGTCTCGACCATATGAGGGAAGTACTTGGAGATGCTGTGCCAGATGAAATATTAATTGAAGCAGTTCTGAAGAACAAGTTTGATGTGCAGAAGGCTTTGTCAGGGGTTCTGGAACAAGATAGAGTGCAGAGTTTGAAGGACAAGAATGAGGCAACAGTATCTACAGGAAAGATAGCAAAAGGAAAACCAGTAGATTCCCAGACATCGCGAAGTGAATCTGAAATTGTGCCAAAAGTTGCTAAAATGACTGTATCTGGAAAGAAGCAAACTATGGGATTTGAAGTGCCTGGAGTATCTTCTGAAGAAAATGGTCATAGTTTCCACACACCTCAAAAAGGACCGCCCATTGAAGATGCCATTGCTTCTTCCGATGTTCTTGAGACTGCTTCTAAATCTGCTAATCCACCCCACACGATTCAAGCATCAGAAGAGCAGAGTTCAACCCCAGCACCGGTGAAAAAGTCTGGCAAGCTGAGGCAGCAAATAGATGTGAAGGCGGAACTGGAGAAGCGGCAAGGAGGGAAGCAGCTACTCAACTTAGTGGTCATTGGTCATGTTGATGCTGGGAAAAGTACTCTGATGGGCCATATGCTTTATCTTCTGGGTAATATAAACAAAAGAACTATGCATAAGTATGAACAGGAGTCTAAAAAGGCTGGCAAAGCTTCGTTTGCATATGCATGGGTCTTGGATGAAACTGGCGAAGAAAGGGAGAGAGGCGTCACAATGGATGTTGGTATGACAAAGTTTGAAACCACAACCAAAGTTATTACATTAATGGATGCTCCAGGCCATAAGGACTTCATTCCAAATATGATTACAGGAGCAGCCCAGGCGGATGTAGCTGTTTTAGTTGTAGATGCCAGCAGGGGAGAGTTTGAAGCTGGATTTGAGACTGGAGGACAAACACGAGAGCATGGACTCTTGGTCCGTTCTCTGGGAGTGACGCAGCTTGCAGTTGCAGTTAATAAAATGGATCAGGTTAATTGGCAACAAGAAAGGTTTCAAGAGATTACTGGAAAACTTGGGCACTTTCTTAAGCAAGCAGGTTTTAAGGAGAGTGATGTAGGTTTTATTCCTACAAGTGGTCTCAGTGGTGAAAATCTAATCACAAGATCTCAGTCAAGTGAACTCACAAAATGGTATAAAGGACTATGTTTATTAGAACAAATTGATTCCTTTAAGCCTCCCCAGCGATCTATTGACAAACCTTTTAGATTATGTGTGTCCGATGTTTTCAAAGATCAAGGATCTGGATTTTGCATAACTGGTAAAATAGAAGCTGGTTATATCCAAACTGGTGACCGACTACTGGCAATGCCTCCTAATGAAACTTGTACCGTGAAAGGAATCACTCTGCATGATGAACCTGTCGACTGGGCGGCAGCAGGCGATCATGTTAGTCTTACTTTGGTTGGGATGGATATCATCAAAATCAATGTTGGCTGCATATTTTGTGGCCCCAAAGTACCCATTAAAGCTTGCACTCGTTTCAGAGCCCGAATCCTCATCTTTAATATTGAAATTCCTATCACTAAAGGATTTCCTGTGCTGTTACACTACCAAACTGTCAGTGAACCCGCCGTTATTAAACGATTGATTAGTGTCTTAAACAAAAGCACGAATGAAGTCACAAAGAAAAAGCCTAAGTTTTTGACTAAAGGCCAGAATGCATTGGTAGAGCTACAGACACAAAGACCAATAGCTCTTGAGCTATATAAAGACTTTAAAGAGCTGGGGAGGTTCATGCTACGTTACGGTGGTTCTACAATAGCTGCTGGTGTTGTCACTGAGATAAAAGAATGA |
| pTG5238 | GSPT1 G575N | GATCCGGGCAGTGGAGGCGGAGGAGGCGGCGGAGGCGGAGGCGGGAGCAGCAGCGGCAGCAGCAGCAGCGACTCGGCGCCTGACTGCTGGGACCAGGCGGACATGGAAGCCCCCGGGCCGGGCCCTTGCGGCGGCGGCGGCTCCCTGGCGGCGGCGGCCGAGGCCCAGCGGGAGAACCTCAGCGCGGCCTTCAGCCGGCAACTCAACGTCAACGCCAAGCCCTTCGTGCCCAACGTCCACGCCGCCGAGTTCGTGCCGTCCTTCCTGCGGGGCCCGGCAGCGCCGCCACCCCCAGTTGGCGGCGCCGCCAATAACCACGGAGCCGGCAGCGGCGCGGGAGGCCGTGCGGCACCTGTGGAATCCTCTCAAGAGGAACAGTCATTGTGTGAAGGTTCAAATTCAGCTGTTAGCATGGAACTTTCAGAACCTATTGTAGAAAATGGAGAGACAGAAATGTCTCCAGAAGAATCATGGGAGCACAAAGAAGAAATAAGTGAAGCAGAGCCAGGGGGTGGTTCCTTGGGAGATGGAAGGCCGCCAGAGGAAAGTGCCCATGAAATGATGGAGGAGGAAGAGGAAATCCCAAAACCTAAGTCTGTGGTTGCACCGCCAGGTGCTCCTAAGAAAGAGCATGTAAATGTGGTTTTTATCGGACATGTAGATGCTGGCAAGTCAACCATTGGAGGACAAATAATGTATTTGACTGGAATGGTTGACAAAAGGACGCTTGAAAAGTATGAAAGAGAAGCTAAAGAGAAAAACAGAGAAACTTGGTACTTGTCTTGGGCCTTAGACACAAATCAGGAAGAACGAGACAAGGGTAAAACAGTAGAAGTGGGTCGTGCCTATTTTGAAACCGAAAAGAAGCATTTCACAATTCTAGATGCCCCTGGCCACAAGAGTTTTGTCCCAAATATGATTGGTGGTGCCTCTCAAGCTGATTTGGCTGTGCTGGTAATCTCAGCCAGGAAAGGAGAGTTTGAAACTGGATTTGAAAAAGGAGGACAGACAAGAGAACATGCTATGCTTGCTAAAACTGCAGGTGTAAAACACCTAATTGTGCTAATTAATAAGATGGATGATCCAACAGTAAATTGGAGCAATGAGAGATATGAAGAATGTAAGGAGAAACTAGTGCCATTTTTGAAAAAAGTTGGCTTCAATCCCAAAAAGGACATTCACTTTATGCCCTGCTCAGGACTTACTGGAGCAAATCTCAAAGAGCAGTCGGATTTCTGTCCTTGGTACATTGGATTACCGTTTATTCCATATCTGGATAATTTGCCGAACTTCAATAGAAGTGTGGACGGGCCTATTAGACTGCCAATTGTGGATAAGTACAAGGATATGGGCACTGTGGTCCTGGGAAAGCTGGAATCAGGATCTATTTGTAAAGGCCAGCAGCTTGTGATGATGCCAAACAAGCACAACGTGGAAGTTCTTGGAATACTTTCCGATGATGTAGAGACTGATACCGTAGCCCCAGGTGAAAACCTCAAAATCAGACTGAAAGGAATTGAAGAAGAGGAGATTCTTCCAGGGTTTATACTTTGTGATCCTAATAATCTTTGTCATTCTGGACGCACATTTGATGCCCAGATAGTGATTATAGAGCACAAATCCATCATCTGCCCAGGCTATAATGCGGTGCTGCATATTCATACCTGTATTGAGGAGGTGGAAATAACAGCCTTAATCTGCTTGGTAGACAAAAAATCAAACGAAAAAAGTAAGACCCGACCCCGTTTTGTCAAACAAGATCAAGTATGCATTGCTCGCTTAAGGACAGCAGGAACCATCTGCCTTGAGACCTTTAAAGACTTCCCTCAGATGGGTCGTTTCACCTTAAGAGATGAGGGTAAGACCATTGCAATTGGAAAAGTTCTGAAACTGGTTCCAGAGAAAGACTAA |
