# Supplementary material for: TNG961 Is a Selective Oral HBS1L Molecular Glue Degrader for the Treatment of FOCAD-Deleted Cancers
Source: Cancer Discov. 2026 Apr 19;16(9):1884–902. doi: 10.1158/2159-8290.CD-26-0040 (PMC13531002; doi:10.1158/2159-8290.CD-26-0040)
Supplement: Supplementary file 10 [file cd-26-0040_table_s4_suppst4.docx]

**Supplementary Table S4. Cryo-EM data collection, refinement, and validation statistics**

|  | HBS1L/CRBN/DDB1  + TNG-4857  (EMD-75840) (PDB 11MR) | | HBS1L/CRBN/DDB1  + TNG961  (EMD-75038) (PDB 10AY) | |
| --- | --- | --- | --- | --- |
| Data collection and processing |  | |  | |
| Magnification | 130,000 | | 130,000 | |
| Voltage (kV) | 300 | | 300 | |
| Electron exposure (e^-^/Å^2^) | 48 | | 48 | |
| Exposure time (s) | 4.83 | | 4.89 | |
| Number frames per movie | 1602 (eer frame) | | 42 | |
| Defocus range (mm) | -1.0~-2.0 | | -1.0~-2.2 | |
| Pixel size (Å) | 0.932 | | 0.932 | |
| Number of movies | 16,029 | | 9532 | |
| Symmetry imposed | C2 | | C2 | |
| Initial particle images (No.) | 10,420,085 | | 4,475,226 | |
| Final particle images (No.) | 661,458 | | 669,259 | |
| Map resolution (Å) | 2.6 | | 2.9 | |
| FSC threshold | 0.143 | | 0.143 | |
| Map resolution range (Å) | 2.6-3.3 | | 2.8~6.3 | |
| Model Refinement |  | |  | |
| Initial model used (PDB codes) | 5HXB | | 5HXB | |
| Map sharpening B factor (Å^2^) | -62.2 | | -88.3 | |
| Model composition |  | |  | |
| Non-hydrogen atoms | 7798 | | 20,140 | |
| Protein residues | 962 | | 2524 | |
| Ligands | 2 | | 2 | |
| B factors (Å^2^) |  | |  | |
| Protein | 63.9 | | 35.9 | |
| Ligand | 36.4 | | 16.9 | |
| R.m.s. deviations |  | |  | |
| Bond lengths (Å) | 0.004 | | 0.004 | |
| Bond angles (°) | 0.560 | | 0.552 | |
| Validation |  | |  | |
| MolProbity score | 1.51 | | 1.64 | |
| Clashscore | 3.64 | | 5.50 | |
| Rotamer outliers (%) | 1.39 | | 0.49 | |
| Ramachandran plot |  | |  | |
| Favored (%) | 96.28 | | 95.06 | |
| Allowed (%) | 3.72 | | 4.85 | |
| Outliers (%) | 0 | | 0.08 | |
|  | |  | |  |
